# Analysis of Surface Immunoglobulin Expression in Burkitt Lymphoma Reveals a Subset of IgA-Expressing Cases Enriched at Mucosal Sites and Surface Immunoglobulin-Undetectable Cases: Insights into the Mutational Landscape

**DOI:** 10.3390/cancers18152453

**Published:** 2026-07-30

**Authors:** Maria Chiara Siciliano, Cosimo Lori, Margherita Vannucchi, Teresa Amato, Giorgio Bertolazzi, Raffaella Guazzo, Onyango Noel, Timothy Onyuma, Massimo Granai, Roberto Boccacci, Kiraka Grace, Oyiro Peter, Simon Onsongo, Magoma Georgina, Sakeah Patience Wedaga, Kwawu Foster, Hagembe Mildred, Nyagol Joshua, Anja Fischer, Cristiana Bellan, Reiner Siebert, Lorenzo Leoncini, Stefano Lazzi

**Affiliations:** 1 Department of Medical Biotechnologies, University of Siena, 53100 Siena, Italy; siciliano10@student.unisi.it (M.C.S.); c.lori1@student.unisi.it (C.L.); margherita.vannucchi@ao-siena.toscana.it (M.V.); teresa.amato@unisi.it (T.A.); raffaella.guazzo@ao-siena.toscana.it (R.G.); massimo.granai@unisi.it (M.G.); roberto.boccacci@student.unisi.it (R.B.); cristiana.bellan@unisi.it (C.B.); lazzi2@unisi.it (S.L.); 2 Department of Medicine and Surgery, Kore University of Enna, 94100 Enna, Italy; giorgio.bertolazzi@unikore.it; 3 Department of Medical Microbiology and Immunology, University of Nairobi, Nairobi 00202, Kenya; noelo@uonbi.ac.ke (O.N.); dronyuma@gmail.com (T.O.); g.kiraka@student.unisi.it (K.G.); 4 KAVI-Institute for Clinical Research (KAVI-ICR), University of Nairobi, Nairobi 00202, Kenya; 5 Department of Clinical Medicine and Therapeutics, University of Nairobi, Nairobi 00202, Kenya; peteroyiro@uonbi.ac.ke (O.P.); georginamagomaedesa@students.uonbi.ac.ke (M.G.); sakeahpatience@students.uonbi.ac.ke (S.P.W.); fosterkwawu@students.uonbi.ac.ke (K.F.); mildredhagembe@mtrh.go.ke (H.M.); 6 Department of Pathology, Aga Khan Hospital Kisumu, Kisumu 40100, Kenya; simonya@live.com; 7 Department of Human Pathology, University of Nairobi, Nairobi 00202, Kenya; joshua.nyagol@uonbi.ac.ke; 8 Institute of Human Genetics, Ulm University and Ulm University Medical Center, 89081 Ulm, Germany; anja.fischer@uni-ulm.de (A.F.); reiner.siebert@uni-ulm.de (R.S.); 9 Center of Rare Diseases (ZSE) Ulm, 89081 Ulm, Germany; 10 German Center for Child and Adolescent Health (DZKJ), Partner Site Ulm, 89081 Ulm, Germany

**Keywords:** Burkitt lymphoma, B-cell receptor, surface immunoglobulin, immunohistochemistry, IgA, surface immunoglobulin-undetectable, sIg-UND, mutational landscape

## Abstract

**Background/Objectives**: B-cell receptor (BCR) signalling is implicated in Burkitt lymphoma (BL) lymphomagenesis, although its activation may differ across biological and epidemiological settings. While surface IgM is the predominant immunoglobulin isotype of BCR in BL, cases expressing IgA transcripts or lacking detectable surface immunoglobulin (sIg) expression have been reported, suggesting a more complex pattern of sIg expression than previously recognized. This study aimed to evaluate sIg heavy-chain expression by immunohistochemistry in a relatively large series of BL cases from endemic and sporadic settings and to investigate the mutational landscape of surface immunoglobulin-undetectable (sIg-UND) cases. **Methods**: sIg heavy-chain expression was assessed by immunohistochemistry in 55 formalin-fixed paraffin-embedded BL samples. Targeted next-generation sequencing was performed on four sIg-UND cases using an Illumina capture-based custom panel covering 74 lymphoma-related genes. *KRAS* and *NRAS* hotspot mutations were additionally assessed by real-time PCR. **Results**: Overall, 41/55 cases showed IgM expression, including 31 IgM-positive and 10 IgM+/UND cases, whereas 8/55 cases showed IgA expression (IgA+/UND). IgA-expressing cases were significantly enriched at mucosal sites, particularly the oral cavity and gastrointestinal tract, consistent with the relevance of these anatomical sites to mucosal IgA production. Six cases lacked detectable sIg expression, showing negativity for all tested immunoglobulin heavy chains in more than 90% of neoplastic cells. Four sIg-UND cases were available for sequencing. These cases harbored mutations affecting genes commonly altered in BL, together with alterations in genes less commonly represented in recurrent BL series, such as *MEF2B*, *CREBBP*, *PRDM1*, *PIM1*, *ARID3A*, *HIST1H1B*, and *HIST1H1C*. **Conclusions**: Our study provides a systematic immunohistochemical characterization of surface immunoglobulin heavy-chain expression in a relatively large series of endemic and sporadic BL. We identified a subset of IgA-expressing BL cases enriched at mucosal sites, whereas rarer cases lacked detectable surface immunoglobulin expression. The mutational profile of sIg-UND cases highlights alterations affecting genes involved in epigenetic regulation, chromatin organization, and B-cell differentiation, which may contribute to the biological heterogeneity of these cases. No *KRAS* or *NRAS* hotspot mutations were detected in the four sequenced sIg-UND cases, as assessed by targeted NGS and real-time PCR. Further functional studies are needed to clarify the biological and clinical significance of sIg-UND BL cases and to determine the functional relevance of the identified alterations.

## Simple Summary

B-cell receptor signalling is an important driver of lymphomagenesis, although its role in Burkitt lymphoma may vary across different biological settings. In this study, we performed immunohistochemical analysis in a cohort of 55 Burkitt lymphoma cases and found that most cases expressed IgM, whereas a subset showed IgA expression and was significantly enriched at mucosal sites, while a few cases lacked detectable surface immunoglobulin expression. Surface immunoglobulin-undetectable cases were then analyzed by next-generation sequencing, revealing mutations less commonly reported in Burkitt lymphoma, which may reflect biological heterogeneity in this rare subset.

## 1. Introduction

The B-cell receptor (BCR) is essential for normal B-cell development and maturation. A functional BCR complex includes membrane immunoglobulin and the signaling subunits CD79A/CD79B, which enable downstream signaling. There is increasing evidence that BCR signalling is implicated in B-cell malignancies [1,2]. A role for BCR signalling in lymphoma has been inferred by studying immunoglobulin genes in human lymphomas and by engineering mouse models [3]. Among B-cell malignancies, multiple mechanisms can lead to activation of BCR signalling. Roles for antigen-dependent and antigen-independent BCR signalling have now been described across several lymphoma types [4,5]. In BL, different mechanisms of BCR activation may operate.

In EBV-negative BL, which more commonly occurs in sporadic/non-endemic settings, recurrent mutations in the *TCF3* and *ID3* genes result in intrinsic tonic activation of BCR signalling [6]. Mechanistically, TCF3 functions as a master regulator of tonic BCR signalling, promoting continuous PI3K/AKT activation and cell survival. Mutations in *ID3* abolish its inhibitory interaction with TCF3, resulting in constitutive TCF3 activity, enhanced immunoglobulin gene transcription, and sustained surface BCR expression. Concurrently, TCF3-mediated repression of *PTPN6* (encoding SHP-1), a negative regulator of BCR signalling, removes inhibitory feedback and establishes a self-reinforcing BCR-PI3K signalling loop. This loop sustains proliferation and metabolic reprogramming, which are molecular features of the Burkitt lymphoma phenotype [6,7,8].

However, genomic studies demonstrate that EBV-positive endemic BL displays a distinct mutational spectrum, characterized by an almost mutual exclusivity between *TCF3* mutations and EBV positivity, suggesting a different pathogenetic mechanism [9,10,11]. Indeed, NGS-based BCR clonality and repertoire analyses in EBV-positive BL revealed evidence of antigen-selected BCR repertoires, consistent with antigen-driven selection that could potentially be related to prolonged microenvironmental interactions [12,13,14]. For African BL, this may be linked to chronic *Plasmodium falciparum* malaria stimulation, which may reactivate latently EBV-infected memory B cells. Yet, whether coincidental infections with additional pathogens may occur in BL remains to be addressed [11,15,16,17]. Nevertheless, a combination of tonic and extrinsic BCR signalling activation cannot be excluded in a subset of BL.

Surface IgM is the predominant immunoglobulin isotype expressed in BL and generally represents the membrane immunoglobulin component of the B-cell receptor complex. However, evidence of IgA expression in BL has occasionally been reported. In particular, an anecdotal work of Chapman et al. [18] identified an IgA-expressing EBV-negative sporadic BL-derived cell line. More recently, genomic and transcriptomic analyses identified, in a subset of BL cases, a significant skewing of *IGH*::*MYC* breakpoints toward *IGHA* switch regions, associated with higher *IGHA* transcript expression [19,20]. However, these findings were not complemented by the assessment of IgA protein expression or by a direct correlation with anatomical localization and EBV status. In addition, rare BL cases with partial or complete loss of detectable surface immunoglobulin expression in most of the neoplastic cells have been reported, suggesting impaired BCR function [21,22]. However, a recent study demonstrated that BCR ablation in MYC-driven mouse B-cell lymphomas does not significantly affect lymphoma growth [22]. In this experimental model, BCR loss was associated with rewiring of central carbon metabolism, increasing the sensitivity of receptor-less lymphoma cells to nutrient restriction [22]. BCR-negative tumor cells were shown to restore competitive fitness by increasing GSK3β activity after constitutive activation of the MAPK pathway, commonly through *RAS* mutations. Interestingly, heterozygous hotspot *RAS* mutations were identified both in the murine experimental model and in a primary BL tumor bearing a substantial fraction of IgM-negative malignant cells. These findings support the hypothesis that BL cells acquiring *RAS* mutations may lose selective pressure to express a functional BCR [22].

Altogether, these findings suggest that sIg expression in BL may be more complex than previously appreciated, prompting us to perform an immunohistochemical analysis in a relatively large cohort of BL cases from endemic and sporadic regions to better define the status of sIg expression in relation to anatomical site and EBV status. We also explored the mutational landscape of surface immunoglobulin-undetectable (sIg-UND) cases.

## 2. Materials and Methods

Case selection: The cohort consisted of Formalin-Fixed Paraffin-Embedded (FFPE) samples from patients diagnosed with BL (Supplementary Table S1) which were retrospectively retrieved from the Department of Medical Biotechnologies, University of Siena (Siena, Italy), and the University of Nairobi (Nairobi, Kenya). The diagnosis of BL was confirmed in 55 cases, by expert haematopathologists (L.L., S.L. and T.O.), according to the criteria outlined in the 5th edition of the World Health Organization (WHO) Classification of Haematolymphoid Tumours. Additionally, medical records were reviewed for sex, age, clinical presentation, EBV status, and HIV infection status.

Immunohistochemistry (IHC): Diagnostic immunohistochemistry was performed in all cases at the University of Siena, using a large panel of antibodies including CD20 (L26—Roche Diagnostics, Monza, Italy), CD10 (SP67—Roche Diagnostics), CD38 (SP149—Cell Marque, Rocklin, CA, USA), BCL6 (GI191E/A8—Roche Diagnostics), LMO2 (SP51—Merck, Darmstadt, Germany), BCL2 (124—Roche Diagnostics), c-MYC (Y69—Roche Diagnostics), and Ki67 (30-9—Roche Diagnostics). In addition, antibodies against immunoglobulin heavy chains were used to evaluate heavy-chain protein expression, including IgG (Polyclonal—Merck), IgA (EP170—Cell Marque), IgM (Polyclonal—Merck), IgE (RM122—Merck) and IgD (EP173—Cell Marque). Staining was performed on the Ventana BenchMark ULTRA (Roche Diagnostics), according to the manufacturer’s instructions. Cases with detectable IGH expression in more than 90% of tumor cells were classified as IGH-positive. Cases showing detectable IGH expression in 10% to 90% of tumor cells were classified as positive/undetectable (+/UND). Conversely, cases in which more than 90% of tumor cells lacked detectable IGH reactivity were classified as surface immunoglobulin-undetectable (sIg-UND) [23]. For isotype-specific analyses, the term “IgM-positive” refers to cases with IgM expression in more than 90% of tumor cells, whereas “IgM-expressing” includes both IgM-positive and IgM+/UND cases, while “IgA-expressing” refers to IgA+/UND cases. To assess EBV status, in situ hybridization for EBV-encoded small RNAs was performed on 5 μm thick FFPE sections from all cases, using an automated staining system (Ventana BenchMark ULTRA, Roche Diagnostics), as previously described.

Fluorescence in situ hybridization (FISH): Fluorescence in situ hybridization analysis was performed in all cases to assess *MYC* rearrangement (Vysis *MYC* Dual Color Break Apart Rearrangement Probe; Abbott, Wiesbaden, Germany), *MYC*::*IGH*/t(8;14) rearrangement (*MYC*::*IGH*, ZytoVision GmbH, Bremerhaven, Germany), and *MYC*::*IGL*/t(8;22) and *MYC*::*IGK*/t(2;8) rearrangements (probes generated by the Institute of Human Genetics, Ulm University Medical Center, Ulm, Germany) [24]. *BCL6* and *BCL2* rearrangements were investigated in all cases using the corresponding break-apart probes (Vysis *BCL2* and *BCL6* Dual Color Break Apart Rearrangement Probes; Abbott, Wiesbaden, Germany), according to the manufacturer’s instructions. Furthermore, FISH was performed in all cases to assess aberrations on the long arm of chromosome 11q (ZytoLight SPEC 11q gain/loss Triple Color Probe).

DNA extraction and processing before next-generation sequencing (NGS): Total DNA from four sIg-UND FFPE samples was extracted from 5 μm thick sections using the MagCore^®^ Automated Nucleic Acid Extraction system (RBC Bioscience, New Taipei City, Taiwan), following the manufacturer’s instructions. Briefly, the procedure is based on magnetic bead technology and includes deparaffinization, proteinase K digestion, DNA extraction, and elution. DNA quantity and quality were evaluated by measuring the optical density (OD) at 260 nm and the 260/230 and the 260/280 ratios using a NanoDrop spectrophotometer (ND-100, NanoDrop, Thermo Scientific, Waltham, MA, USA). DNA concentration was further measured using the Qubit™ dsDNA HS Assay Kit (Qubit 4, Thermo Fisher Scientific, Waltham, MA, USA), following the manufacturer’s instructions. The fragmentation level of extracted tumor DNA was assessed before library preparation using the QIAxcel Advanced System, based on capillary electrophoresis (Qiagen, Hilden, Germany).

Library preparation and sequencing: For next-generation sequencing (NGS), libraries were prepared from extracted DNA using approximately 100 ng of DNA per sample with the Illumina DNA Prep with Enrichment Kit and a custom targeted sequencing panel (Illumina, San Diego, CA, USA) covering 74 lymphoma-related genes. Library size distribution and quality were assessed using the QIAxcel Advanced System, based on capillary electrophoresis (Qiagen, Hilden, Germany). Sequencing libraries were quantified using the Qubit™ dsDNA High Sensitivity Assay Kit (Thermo Fisher Scientific, Waltham, MA, USA). The prepared libraries were then loaded onto the Illumina MiSeq platform using the MiSeq Reagent Kit V2 for a paired-end 2 × 151-cycle run. Binary Alignment Map (BAM) files were generated using Illumina Local Run Manager by aligning sequencing reads to the GRCh37/hg19 reference genome. Following alignment, variants were called using Illumina Local Run Manager. Variant annotation was subsequently performed using ANNOVAR (https://annovar.openbioinformatics.org/, accessed on 29 July 2026). Variants with a VAF below 2% were excluded. Relevant tumor-derived variants were called and filtered for downstream analyses according to the following criteria: (I) location within an exonic or splicing region; (II) non-synonymous effect; and (III) median target coverage depth >300×. All variants found in the ClinVar database and classified as germline variants, or common population single-nucleotide polymorphisms (SNPs), were excluded. Given the use of FFPE-derived DNA, candidate variants fulfilling these criteria were manually inspected in Integrative Genomics Viewer (IGV) to evaluate read support, strand distribution, local sequence context, and features suggestive of sequencing or FFPE-related artifacts. Variants considered technically unreliable were excluded from downstream interpretation. Variants were considered relevant when previously reported in COSMIC, Franklin, or VarSome, or when affecting the same functional domain as known pathogenic alterations. Prediction tools, including PolyPhen (http://genetics.bwh.harvard.edu/pph2/, accessed on 29 July 2026) and SIFT (https://sift.bii.a-star.edu.sg/, accessed on 29 July 2026), were used to support the prediction of variant impact. The variants identified in our four sIg-UND BL cases were then interpreted according to whether the affected genes are commonly mutated in BL [20], diffuse large B-cell lymphoma (DLBCL) [25], or high-grade B-cell lymphoma, not otherwise specified (HGBL-NOS) [26,27], based on the selected reference datasets. Genes not clearly assignable to one of these categories based on the selected reference datasets were considered unclassified.

Real-time PCR: Hotspot mutations in both *KRAS* and *NRAS* were further assessed by real-time PCR using commercially available allele-specific assays (EasyPGX^®^, Diatech Pharmacogenetics, Jesi, Italy), according to the manufacturer’s instructions. The analysis was performed on DNA from the four sIg-UND cases.

## 3. Results

### 3.1. Clinical and Pathological Characteristics of the BL Cohort

The cohort included 40 cases (73%) of BL from endemic regions and 15 cases (27%) of sporadic BL. The clinicopathological characteristics of the cases are summarized in Supplementary Table S1. In particular, our cohort consisted of 34 male (62%) and 21 female (38%) patients. Epstein–Barr virus infection was found in 39 cases (71%) of our cohort, 28 (72%) of which belonged to the endemic group. The median age at diagnosis was 28 years (range, 3–78 years). Anatomical localization of eBL included the gastrointestinal tract, oral cavity, kidney, ovary, lymph nodes, and abdominal masses, while anatomical localization of sBL included bone marrow and the gastrointestinal tract. All BL cases were centrally reviewed and independently confirmed by three expert hematopathologists according to the criteria outlined in the 5th edition of the WHO Classification of Haematolymphoid Tumours. All cases showed the typical morphology and immunophenotype of BL (CD20+, CD10+, BCL6+, LMO2−, CD38+, BCL2−, MYC > 80%, and Ki67 > 95%). Fluorescence in situ hybridization demonstrated an isolated *MYC* rearrangement in all cases: an *IGH*::*MYC* translocation was detected in 51/55 cases (93%), whereas *MYC*::*IGL* or *MYC*::*IGK* translocations were identified in the remaining 4/55 cases (7%). No *BCL2* or *BCL6* rearrangements or 11q aberrations were detected (Supplementary Table S1).

### 3.2. IgA-Expressing BL Cases Are Enriched at Mucosal Sites

**Figure 1:**
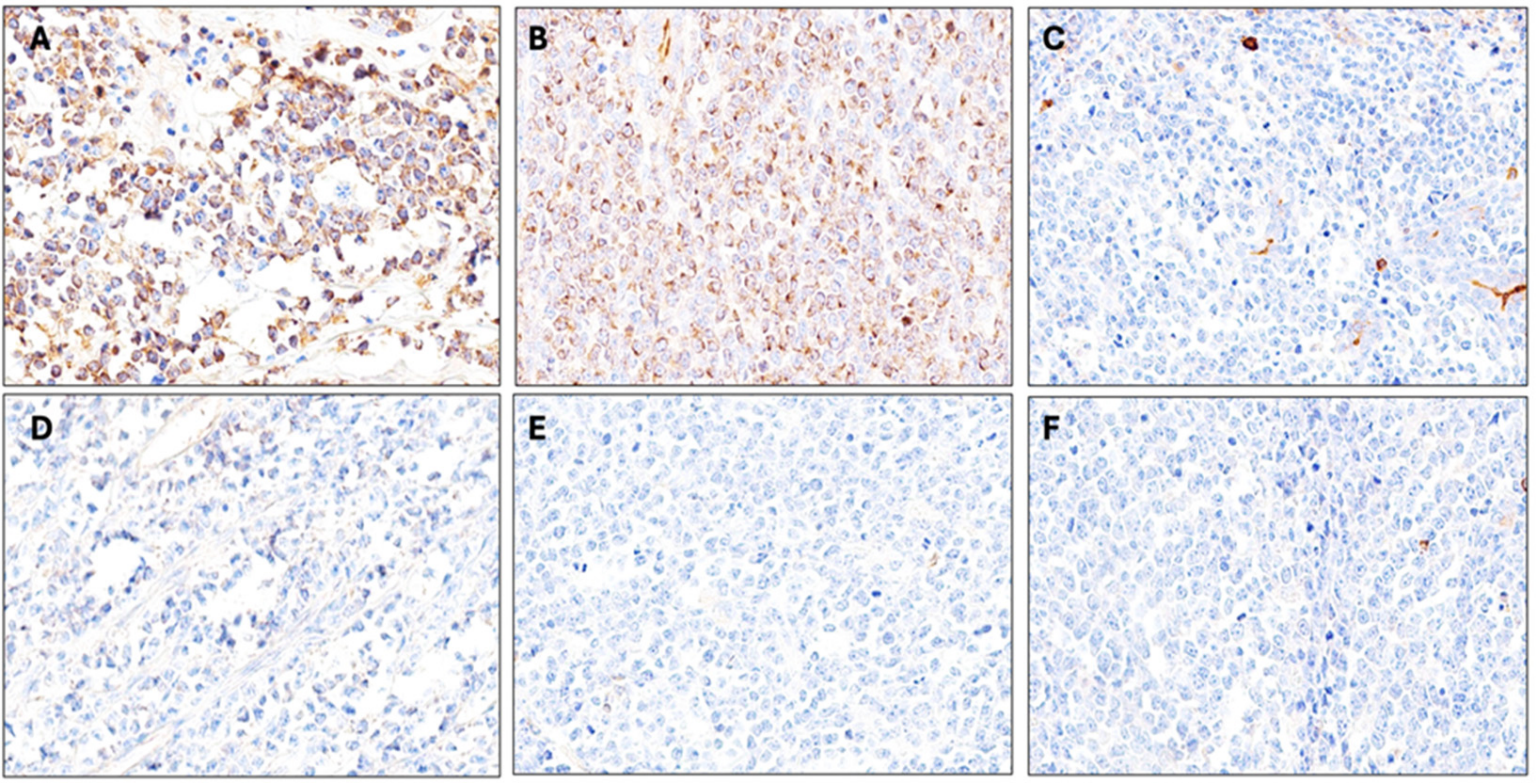
Immunohistochemical assessment of IgA and IgM expression in BL cases. Representative immunohistochemical staining for IgA and IgM. Panels (**A**,**D**) show an IgA-expressing BL case, with IgA positivity in A and absence of IgM staining in (**D**). Panels (**B**,**E**) show an IgM-positive BL case, with IgM positivity in (**B**) and absence of IgA staining in (**E**). Panels (**C**,**F**) show the absence of IgA and IgM staining, respectively, in an sIg-UND BL case.

Immunohistochemical analysis of immunoglobulin heavy-chain expression was performed (Figure 1), and cases were classified as described in the Section 2. Among the 55 BL cases, no cases showed expression of IgG, IgE, or IgD. In contrast, 31 cases (56%) were classified as IgM-positive, showing IgM expression in more than 90% of tumor cells. Among the remaining 24 cases, 10 (18%) were classified as IgM+/UND, and 8 cases (15%) as IgA+/UND, showing surface immunoglobulin expression in 10% to 90% of tumor cells. Only 6 cases (11%) were classified as sIg-UND, as more than 90% of tumor cells lacked detectable reactivity for all tested immunoglobulin heavy chains.

Interestingly, IgA-expressing cases showed a trend toward enrichment among African cases, although this association was not statistically significant (*p* = 0.29), while they were significantly enriched at mucosal sites, such as the oral cavity and gastrointestinal tract (*p* = 0.028). EBV positivity was detected in 3 of 6 sIg-UND cases (50%), in 6 of 8 IgA-expressing cases (75%), and in 30 of 41 IgM-expressing cases (73%). However, no significant association was observed between EBV and HIV status and surface immunoglobulin expression pattern.

### 3.3. Surface Immunoglobulin-Undetectable BL Cases Show a Heterogeneous Mutational Landscape with Uncommon Alterations

**Figure 2:**
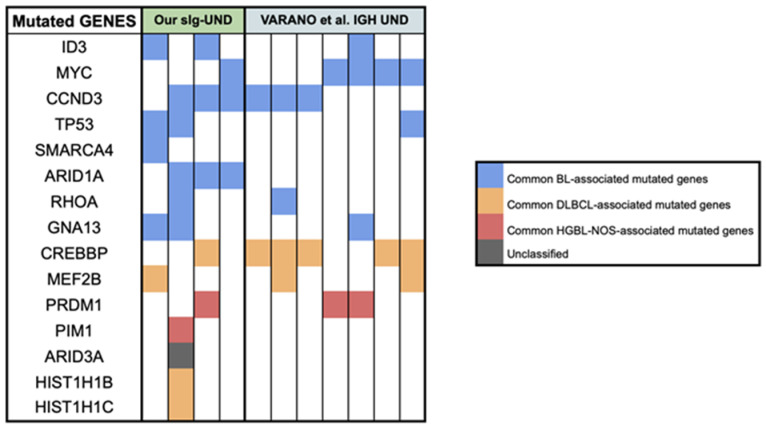
Comparative oncoplot of sIg-UND BL cases. Genes shown in the first column were selected based on the alterations identified in our four sIg-UND BL cases and compared with the IGH-UND cohort reported by Varano et al. [23]. Gene alterations were color-coded according to their relative representation in selected reference datasets [20,25,26,27]: blue, BL-associated; orange, DLBCL-associated; red, HGBL-NOS-associated; gray, unclassified. This classification refers to the selected datasets and does not imply a distinct diagnostic entity.

To investigate the mutational landscape of our sIg-UND BL cases, we performed targeted NGS in four cases with available DNA using a custom panel of 74 genes involved in B- and T-cell lymphomas. As shown in Figure 2, these four cases harbored alterations in genes commonly mutated in BL, including *ID3*, *MYC*, *CCND3*, *TP53*, *SMARCA4*, *ARID1A*, *RHOA*, and *GNA13* [20]. Alongside these, in three out of four cases, we identified additional alterations in genes less commonly represented in recurrent BL series, including *CREBBP*, *MEF2B*, *HIST1H1B*, and *HIST1H1C*, which are more frequently reported in DLBCL datasets [25], together with *PRDM1* and *PIM1*, which are more frequently reported in HGBL-NOS datasets [26,27]. These associations refer to their relative representation in the selected reference datasets and do not imply a DLBCL-like or HGBL-NOS-like biological phenotype in our sIg-UND BL cases. *ARID3A* was detected in our series but was not clearly assignable to a distinct entity based on the selected reference datasets and was therefore considered unclassified. A comprehensive overview of the sequencing results, including case-level variant data and associated quality parameters, is provided in Supplementary Tables S2–S4. All four sIg-UND cases were negative for *KRAS* and *NRAS* hotspot mutations, as assessed by both targeted NGS and real-time PCR.

## 4. Discussion

Despite the diversity in the mechanisms driving malignant B-cell transformation, genetic evidence supports the concept that an intact BCR signalling axis plays an important role in the initiation and maintenance of several mature B-cell lymphomas, with classical Hodgkin lymphoma representing a notable exception [8,28]. Specifically, in BL, next-generation sequencing (NGS) studies have revealed recurrent mutations affecting genes involved in or regulating the BCR signalling pathway. Transcription factor 3 (TCF3) is an important component of the tonic BCR signalling pathway, which normally increases the expression of BCR, leading to increased PI3K signalling. This is the most common mechanism observed in EBV-negative BL, resulting in intrinsic activation of BCR signalling [6,20]. Conversely, in EBV-positive BL, BCR activation is mainly sustained by chronic antigenic stimulation, activating B-cell clonotypes with evidence of antigen selection in their BCR repertoire and thereby promoting neoplastic clone growth [11,12]. In BL, IgM is usually the surface immunoglobulin component of the BCR, whereas isotype-switched immunoglobulins are more commonly detected in diffuse large B-cell lymphoma (DLBCL) and follicular lymphoma (FL) [7]. Interestingly, recent genomic studies identified a significant skewing of the *IGH*::*MYC* breakpoints toward *IGHA* switch regions also in a subset of BL cases, with a significantly increased expression of *IGHA* transcripts in sBL [19,20]. Indeed, in the present paper, we confirmed the surface protein expression of IgA in 8 of the 55 cases analyzed. Notably, all of these cases occurred at extranodal sites and were significantly enriched at mucosal sites, particularly in the gastrointestinal tract and oral cavity. This anatomical distribution is of interest, as it is consistent with the possibility that IgA-expressing BL may be related to mucosal immune programs, rather than exclusively to typical IgM-expressing germinal-center (GC) B cells. Previous immunoglobulin gene analyses have shown that BL cells frequently carry mutated *IGHV* genes and may display features consistent with GC or post-GC derivation and antigen-driven selection [29,30]. IgA expression may reflect not only GC transit and class-switch recombination but also the imprinting of these tissues, characterized by differentiation programs linked to mucosal immunity [20,31]. Indeed, these anatomical regions are physiologically enriched in this immunoglobulin isotype [7,20] as a result of chronic antigenic stimulation by pathogens possibly related to these specific sites, including local microbiota-derived antigens [32]. This is in line with the proposed polymicrobial pathogenesis of BL [11,33]. Six of the eight IgA-expressing cases were EBV-positive. However, no statistically significant association between EBV status and immunoglobulin isotype expression was observed in this cohort. Since EBV-positive cases represented the majority of our series, larger studies will be required to determine whether EBV contributes to IgA class switching or whether this distribution simply reflects the underlying epidemiology of the cohort.

We also reported additional BL cases with undetectable surface immunoglobulin expression. Our cases were tested for all heavy chains (IgG-, IgA-, IgM-, IgE- and IgD-), while previous studies did not systematically assess all *IGH* isotypes [21,22].

Targeted NGS analysis of these cases revealed a mutational landscape including alterations in genes commonly altered in BL [34], together with additional mutations in genes less commonly altered in recurrent BL series. Notably, several of these alterations involved genes that are not canonical components of the BCR-associated signalling pathways, such as PI3K/AKT, MAPK/ERK, and NF-κB or the MYC-driven transcriptional program, but are mainly related to epigenetic regulation, chromatin organization, and B-cell differentiation. In particular, mutations involving *CREBBP*, which encodes an acetyltransferase, may affect transcriptional programs and deregulate genes involved in BCR signalling and B cell differentiation, such as *PRDM1*, whose alteration, in turn, may contribute to maintaining tumor cells in a poorly differentiated and proliferative state [35,36,37,38]. Mutations involving *MEF2B* are also noteworthy, as MEF2B is part of the CREBBP-regulated germinal-center transcriptional network and directly transactivates *BCL6*, a key regulator of GC identity [38,39], while *PIM1* mutations may enhance tumor-cell survival [40]. Finally, mutations in linker histone genes, such as *HIST1H1B* and *HIST1H1C,* may alter chromatin structure and promote a more permissive chromatin state [41]. No *KRAS* or *NRAS* hotspot mutations were detected in the four sequenced sIg-UND cases, as assessed by targeted NGS and real-time PCR. Interestingly, mutations of *CREBBP*, *MEF2B* and *PRDM1* were described in a subgroup of high-grade B-cell lymphomas with *MYC* and *BCL2/BCL6* rearrangements (HGBL-DH) and undetectable IGH [23]. Here, we show that alterations affecting the same genes also occur in cases fulfilling the diagnostic criteria for BL and lacking *BCL2* and *BCL6* rearrangements or 11q alterations.

The mechanisms responsible for the lack of detectable surface immunoglobulin expression in sIg-UND BL cases remain to be clarified. Potential explanations include structural or transcriptional alterations of immunoglobulin loci [20,42], impaired assembly or membrane transport of the BCR complex [43], and epigenetic or transcriptional changes affecting B-cell differentiation programs and antibody response maturation [44]. The variants detected in the present study did not primarily involve genes directly related to immunoglobulin loci or core BCR assembly components but rather genes implicated in epigenetic regulation, chromatin organization, and transcriptional regulation of genes involved in BCR signalling and B-cell differentiation [35,39,41]. Such alterations may contribute to tumor-cell fitness through biological programs distinct from canonical surface immunoglobulin-associated signalling axes and from MAPK/ERK and PI3K/AKT signalling activated by *RAS* mutations [22]. However, the functional significance of the variants described here remains unknown, and these findings should be confirmed in additional series and investigated through dedicated functional studies. Therefore, our targeted analysis should be regarded as exploratory and hypothesis-generating. Overall, the present results seem to suggest an alternative uncommon and heterogeneous mutational landscape in BL cases with undetectable surface immunoglobulin expression.

## 5. Conclusions

This work provides a systematic characterization of surface immunoglobulin heavy-chain expression in BL, highlighting a more complex and heterogeneous scenario compared to the traditional view of predominant IgM expression on BL tumor cells. Briefly, in our BL cohort, we identified three different subgroups: a major subgroup expressing IgM, a smaller subgroup expressing IgA, and a few surface immunoglobulin-undetectable cases. Notably, IgA-expressing BL cases were significantly enriched at mucosal sites, particularly in the oral cavity and gastrointestinal tract. In addition, sIg-UND cases are of interest since they are characterized by mutations in genes less frequently altered in recurrent BL series, suggesting a biological heterogeneity of such cases. Although our analysis is limited by the small number of sIg-UND cases and the lack of functional studies, our findings suggest the existence of a biologically intriguing subgroup of BL lacking detectable sIg expression, whose clinical and molecular significance warrants further investigation.

## Data Availability

Data are available on request from co-author, Maria Chiara Siciliano.

## Supplementary Materials

The following supporting information can be downloaded at: https://www.mdpi.com/article/10.3390/cancers18152453/s1, Table S1: Sample cohort with histopathological information, IgA/IgM IHC evaluation, and FISH; Table S2: sIg-UND cases mutations; Table S3: sIg-UND cases sequencing quality metrics; Table S4: Panel target regions.

## Abbreviations

BL	Burkitt lymphoma
BCR	B-Cell Receptor
sIg-UND	Surface immunoglobulin-undetectable
IHC	Immunohistochemistry
FISH	Fluorescence In Situ Hybridization
NGS	Next Generation Sequencing
SNP	Single Nucleotide Polymorphism
EBV	Epstein–Barr Virus
eBL	Endemic Burkitt Lymphoma
sBL	Sporadic Burkitt Lymphoma
DLBCL	Diffuse Large B Cell Lymphoma
FL	Follicular Lymphoma
HGBL-NOS	High Grade B Cell Lymphoma, Not Otherwise Specified
HGBL-DH	High Grade B Cell Lymphoma, Double Hit
GC	Germinal Center
FFPE	Formalin-fixed paraffin-embedded
WHO	World Health Organization
HIV	Human immunodeficiency virus
OD	Optical Density
VAF	Variant allele frequency
