# Supplementary material for: Analysis of Surface Immunoglobulin Expression in Burkitt Lymphoma Reveals a Subset of IgA-Expressing Cases Enriched at Mucosal Sites and Surface Immunoglobulin-Undetectable Cases: Insights into the Mutational Landscape
Source: Cancers (Basel). 2026 Jul 30;18(15):2453. doi: 10.3390/cancers18152453 (PMC13464927; doi:10.3390/cancers18152453)
Supplement: Supplementary file 1 [file cancers-18-02453-s001.zip › cancers-4402736-supplementary.pdf]

cancers

MDPI

Table S1. Sample cohort with histopathological information, IgA/IgM IHC evaluation, and FISH.

Sample ID	IgA	IgM	Gender	Age	Site	EBV Status	Ethnicity	MYC rearrangement	MYC partner	BCL2 rearrangement	BCL6 rearrangement	11q aberration
BL_1	-	-	M	15	Abdomen	+	African (eBL)	Positive	IGH::MYC / t(8;14)	Negative	Negative	Negative
BL_2	-	-	F	6	Gastrointestinal tract	-	Italian (sBL)	Positive	IGH::MYC / t(8;14)	Negative	Negative	Negative
BL_3	-	-	M	5	Oral cavity	+	African (eBL)	Positive	IGH::MYC / t(8;14)	Negative	Negative	Negative
BL_4	-	-	F	3	Kidney	-	African (eBL)	Positive	IGH::MYC / t(8;14)	Negative	Negative	Negative
BL_5	-	-	M	8	Gastrointestinal tract	-	Italian (sBL)	Positive	IGH::MYC / t(8;14)	Negative	Negative	Negative
BL_6	-	-	M	6	Abdominal mass	+	African (eBL)	Positive	IGH::MYC / t(8;14)	Negative	Negative	Negative
BL_7	UND/ +	-	F	63	Gastrointestinal tract	-	African (eBL)	Positive	IGH::MYC / t(8;14)	Negative	Negative	Negative
BL_8	UND/ +	-	M	42	Gastrointestinal tract	+	African (eBL)	Positive	IGH::MYC / t(8;14)	Negative	Negative	Negative
BL_9	UND/ +	-	M	10	Oral cavity	+	African (eBL)	Positive	IGH::MYC / t(8;14)	Negative	Negative	Negative
BL_10	UND/ +	-	M	32	Gastrointestinal tract	-	African (eBL)	Positive	IGH::MYC / t(8;14)	Negative	Negative	Negative
BL_11	UND/ +	-	M	71	Gastrointestinal tract	+	African (eBL)	Positive	IGH::MYC / t(8;14)	Negative	Negative	Negative
BL_12	UND/ +	-	M	12	Oral cavity	+	African (eBL)	Positive	MYC::IGL / t(8;22)	Negative	Negative	Negative
BL_13	UND/ +	-	M	47	Gastrointestinal tract	+	Italian (sBL)	Positive	IGH::MYC / t(8;14)	Negative	Negative	Negative
BL_14	UND/ +	-	M	6	Submandibular mass	+	African (eBL)	Positive	IGH::MYC / t(8;14)	Negative	Negative	Negative
BL_15	-	+	F	9	Oral cavity	+	African (eBL)	Positive	IGH::MYC / t(8;14)	Negative	Negative	Negative
BL_16	-	+	M	12	Oral cavity	+	African (eBL)	Positive	IGH::MYC / t(8;14)	Negative	Negative	Negative
BL_17	-	+	M	73	Lymph node	-	African (eBL)	Positive	IGH::MYC / t(8;14)	Negative	Negative	Negative
BL_18	-	+	F	59	Bone marrow	-	Italian (sBL)	Positive	IGH::MYC / t(8;14)	Negative	Negative	Negative
BL_19	-	+	M	54	Gastrointestinal tract	-	African (eBL)	Positive	IGH::MYC / t(8;14)	Negative	Negative	Negative
BL_20	-	+	F	48	Lymph node	+	African (eBL)	Positive	IGH::MYC / t(8;14)	Negative	Negative	Negative
BL_21	-	+	M	3	Abdominal mass	+	African (eBL)	Positive	IGH::MYC / t(8;14)	Negative	Negative	Negative
BL_22	-	+	F	28	Ovary	+	African (eBL)	Positive	MYC::IGL / t(8;22)	Negative	Negative	Negative

BL_23	-	+	M	71	Bone marrow	+	Italian (sBL)	Positive	IGH::MYC / t(8;14)	Negative	Negative	Negative
BL_24	-	+	M	51	Gastrointestinal tract	+	Italian (sBL)	Positive	IGH::MYC / t(8;14)	Negative	Negative	Negative
BL_25	-	+	M	22	Lymph node	+	African (eBL)	Positive	IGH::MYC / t(8;14)	Negative	Negative	Negative
BL_26	-	+	M	57	Lymph node	-	African (eBL)	Positive	IGH::MYC / t(8;14)	Negative	Negative	Negative
BL_27	-	+	F	26	Lymph node	+	African (eBL)	Positive	IGH::MYC / t(8;14)	Negative	Negative	Negative
BL_28	-	+	F	51	Subcutaneous	+	African (eBL)	Positive	IGH::MYC / t(8;14)	Negative	Negative	Negative
BL_29	-	+	F	32	Lymph node	-	African (eBL)	Positive	IGH::MYC / t(8;14)	Negative	Negative	Negative
BL_30	-	+	F	61	Bone marrow	-	African (eBL)	Positive	IGH::MYC / t(8;14)	Negative	Negative	Negative
BL_31	-	+	F	4	Oral cavity	+	African (eBL)	Positive	IGH::MYC / t(8;14)	Negative	Negative	Negative
BL_32	-	+	F	3	Oral cavity	+	African (eBL)	Positive	IGH::MYC / t(8;14)	Negative	Negative	Negative
BL_33	-	+	F	4	Lymph node	+	African (eBL)	Positive	IGH::MYC / t(8;14)	Negative	Negative	Negative
BL_34	-	+	M	49	Bone marrow	+	Italian (sBL)	Positive	IGH::MYC / t(8;14)	Negative	Negative	Negative
BL_35	-	+	M	55	Bone marrow	+	Italian (sBL)	Positive	IGH::MYC / t(8;14)	Negative	Negative	Negative
BL_36	-	+	M	51	Bone marrow	+	Italian (sBL)	Positive	MYC::IGL / t(8;22)	Negative	Negative	Negative
BL_37	-	+	F	48	Gastrointestinal tract	+	Italian (sBL)	Positive	IGH::MYC / t(8;14)	Negative	Negative	Negative
BL_38	-	+	F	5	Gastrointestinal tract	+	African (eBL)	Positive	IGH::MYC / t(8;14)	Negative	Negative	Negative
BL_39	-	+	M	5	Gastrointestinal tract	-	African (eBL)	Positive	IGH::MYC / t(8;14)	Negative	Negative	Negative
BL_40	-	+	M	14	Abdominal mass	-	African (eBL)	Positive	IGH::MYC / t(8;14)	Negative	Negative	Negative
BL_41	-	+	M	49	Gastrointestinal tract	-	African (eBL)	Positive	MYC::IGK / t(2;8)	Negative	Negative	Negative
BL_42	-	+	M	65	Oral cavity	+	African (eBL)	Positive	IGH::MYC / t(8;14)	Negative	Negative	Negative
BL_43	-	+	M	6	Oral cavity	+	African (eBL)	Positive	IGH::MYC / t(8;14)	Negative	Negative	Negative
BL_44	-	+	M	8	Abdominal mass	+	African (eBL)	Positive	IGH::MYC / t(8;14)	Negative	Negative	Negative
BL_45	-	+	F	10	Submandibular mass	+	African (eBL)	Positive	IGH::MYC / t(8;14)	Negative	Negative	Negative
BL_46	-	UND/+	M	45	Lymph node	+	African (eBL)	Positive	IGH::MYC / t(8;14)	Negative	Negative	Negative
BL_47	-	UND/+	M	37	Gastrointestinal tract	+	Italian (sBL)	Positive	IGH::MYC / t(8;14)	Negative	Negative	Negative
BL_48	-	UND/+	F	26	Gastrointestinal tract	+	Italian (sBL)	Positive	IGH::MYC / t(8;14)	Negative	Negative	Negative

BL_49	-	UND/+	M	69	Bone marrow	-	Italian (sBL)	Positive	IGH::MYC / t(8;14)	Negative	Negative	Negative
BL_50	-	UND/+	M	18	Oral cavity	+	African (eBL)	Positive	IGH::MYC / t(8;14)	Negative	Negative	Negative
BL_51	-	UND/+	M	78	Gastrointestinal tract	+	Italian (sBL)	Positive	IGH::MYC / t(8;14)	Negative	Negative	Negative
BL_52	-	UND/+	M	37	Gastrointestinal tract	+	Italian (sBL)	Positive	IGH::MYC / t(8;14)	Negative	Negative	Negative
BL_53	-	UND/+	F	52	Oral cavity	-	African (eBL)	Positive	IGH::MYC / t(8;14)	Negative	Negative	Negative
BL_54	-	UND/+	F	9	Abdominal mass	+	African (eBL)	Positive	IGH::MYC / t(8;14)	Negative	Negative	Negative
BL_55	-	UND/+	F	3	Abdominal mass	+	African (eBL)	Positive	IGH::MYC / t(8;14)	Negative	Negative	Negative

Table S2. sIg-UND cases mutations.

sIg-UND Case	Gene	NM	Nucleotide Change	Protein Change	VAF%	Read Depth	Mutation Type	Pathogenecity annotation
1	ID3	NM_002167.5	c.190C>T	p.L64F	16.65%	1291x	Missense	VUS
	ID3	NM_002167.5	c.166C>G	p.P56A	5.81%	1312x	Missense	VUS
	TP53	NM_000546.5	c.824G>A	p.C275Y	57.14%	609x	Missense	Pathogenic
	GNA13	NM_006572.6	c.113T>C	p.L38P	10.17%	1288x	Missense	VUS
	GNA13	NM_006572.6	c.48del	p.G17Afs*4	19.29%	1156x	Frameshift deletion	VUS
	SMARCA4	NM_003072.5	c.3575G>A	p.R1192H	26.88%	1099x	Missense	VUS
	MEF2B	NM_001145785.2	c.800del	p.P267Lfs*137	8.82%	340x	Frameshift deletion	VUS
2	ARID1A	NM_006015.6	c.884T>C	p.L295P	7.56%	319x	Missense	VUS
	RHOA	NM_001664.4	c.14G>A	p.R5Q	44.49%	463x	Missense	VUS
	HIST1H1C	NM_005319.4	c.118G>A	p.V40M	49.72%	537x	Missense	VUS
	HIST1H1B	NM_005322.2	c.317G>T	p.G106V	42.92%	480x	Missense	VUS
	PIM1	NM_001243186.2	c.370C>T	p.P124S	39.90%	807x	Missense	VUS
	CCND3	NM_001760.4	c.775T>G	p.S259A	99.70%	668x	Missense	Benign
	TP53	NM_000546.5	c.743G>A	p.R248Q	45.73%	492x	Missense	Pathogenic
	GNA13	NM_006572.6	c.79C>T	p.Q27X	6.90%	652x	Nonsense	VUS
	ARID3A	NM_005224.3	c.418_420del	p.E145del	9.25%	392x	Inframeshift deletion	VUS
3	ID3	NM_002167.5	c.190C>T	p.L64F	36.33%	490x	Missense	VUS
	ID3	NM_002167.5	c.157G>T	p.E53X	39.51%	486x	Nonsense	VUS
	ARID1A	NM_006015.6	c.4915A>T	p.I1639F	53.09%	375x	Missense	VUS
	CCND3	NM_001760.4	c.811dupC	p.R271Pfs*53	30.88%	353x	Frameshift insertion	VUS
	PRDM1	NM_001198.4	c.409A>G	p.R137G	43.44%	344x	Missense	VUS
	CREBBP	NM_004380.3	c.3723C>A	p.F1241L	27.84%	376x	Missense	VUS
4	ARID1A	NM_006015.6	c.2077C>T	p.R693X	2.70%	371x	Nonsense	Pathogenic
	CCND3	NM_001760.4	c.847A>G	p.T283A	2.80%	429x	Missense	VUS
	MYC	NM_002467.6	c.142_144del	p.Q52del	2.94%	476x	Inframeshift deletion	VUS

Table S3. sIg-UND cases sequencing quality metrics.

Case ID	Total reads	Mapped reads (%)	Filtered reads (%)	On-target rate (%)	PCR duplicate fraction (%)	100×	500×	1000×	Coverage 10th percentile	Low-coverage regions
sIg-UND_1	7,456,424	99.19	1.85	89.27	24.30	100	100	98.72	1,574×	1
sIg-UND_2	6,986,774	98.89	2.67	86.79	32.67	100	100	96.49	1,295×	0
sIg-UND_3	7,274,408	99.12	2.12	88.14	28.22	100	100	97.98	1,388×	1
sIg-UND_4	6,485,172	98.51	2.91	84.93	39.73	100	99.7	93.88	1,054×	0

Table S4. Panel target regions.

region_name	seqName	start	stop	region_category	gene	tx_id	tx_version	strand	tx_map	kit_exons	total_exons
TNFRSF14_ex1	1	2487994	2488283	0	TNFRSF14	NM_003820	3	+	ex1	8	8
TNFRSF14_ex2	1	2489055	2489384	0	TNFRSF14	NM_003820	3	+	ex2	8	8
TNFRSF14_ex3	1	2489672	2490017	0	TNFRSF14	NM_003820	3	+	ex3	8	8
TNFRSF14_ex4	1	2491151	2491560	0	TNFRSF14	NM_003820	3	+	ex4	8	8
TNFRSF14_ex5	1	2491953	2492264	0	TNFRSF14	NM_003820	3	+	ex5	8	8
TNFRSF14_ex6	1	2493002	2493378	0	TNFRSF14	NM_003820	3	+	ex6	8	8
TNFRSF14_ex7-8	1	2494194	2494822	0	TNFRSF14	NM_003820	3	+	ex7-8	8	8
ID3_ex1-2	1	23885340	23886029	1	ID3	NM_002167	4	-	ex1-2	0	3
ARID1A_ex1	1	27022783	27024155	0	ARID1A	NM_006015	4	+	ex1	20	20
ARID1A_ex2	1	27056031	27056465	0	ARID1A	NM_006015	4	+	ex2	20	20
ARID1A_ex3	1	27057532	27058206	0	ARID1A	NM_006015	4	+	ex3	20	20
ARID1A_ex4	1	27059057	27059394	0	ARID1A	NM_006015	4	+	ex4	20	20
ARID1A_ex5	1	27087237	27087698	0	ARID1A	NM_006015	4	+	ex5	20	20
ARID1A_ex6	1	27087756	27088074	0	ARID1A	NM_006015	4	+	ex6	20	20
ARID1A_ex7	1	27088532	27088921	0	ARID1A	NM_006015	4	+	ex7	20	20
ARID1A_ex8	1	27089353	27089888	0	ARID1A	NM_006015	4	+	ex8	20	20
ARID1A_ex9-10	1	27092601	27093168	0	ARID1A	NM_006015	4	+	ex9-10	20	20
ARID1A_ex11	1	27094170	27094601	0	ARID1A	NM_006015	4	+	ex11	20	20
ARID1A_ex12	1	27097500	27097928	0	ARID1A	NM_006015	4	+	ex12	20	20
ARID1A_ex13-14	1	27098880	27099589	0	ARID1A	NM_006015	4	+	ex13-14	20	20
ARID1A_ex15-17	1	27099727	27100499	0	ARID1A	NM_006015	4	+	ex15-17	20	20
ARID1A_ex18	1	27100710	27101824	0	ARID1A	NM_006015	4	+	ex18	20	20
ARID1A_ex19	1	27101958	27102309	0	ARID1A	NM_006015	4	+	ex19	20	20
ARID1A_ex20	1	27105404	27107402	0	ARID1A	NM_006015	4	+	ex20	20	20
BCL10_ex3	1	85733308	85733667	1							
BCL10_ex2	1	85736266	85736625	1							
BCL10_ex1	1	85741948	85742067	1							

NRAS_ex5	1	1.15E+08	1.15E+08	0	NRAS	NM_002524	4	-	ex5	4	7
NRAS_ex4	1	1.15E+08	1.15E+08	0	NRAS	NM_002524	4	-	ex4	4	7
NRAS_ex3	1	1.15E+08	1.15E+08	0	NRAS	NM_002524	4	-	ex3	4	7
NRAS_ex2	1	1.15E+08	1.15E+08	0	NRAS	NM_002524	4	-	ex2	4	7
CD58_ex6	1	1.17E+08	1.17E+08	0	CD58	NM_001779	2	-	ex6	6	6
CD58_ex5	1	1.17E+08	1.17E+08	0	CD58	NM_001779	2	-	ex5	6	6
CD58_ex4	1	1.17E+08	1.17E+08	0	CD58	NM_001779	2	-	ex4	6	6
CD58_ex3	1	1.17E+08	1.17E+08	0	CD58	NM_001779	2	-	ex3	6	6
CD58_ex2	1	1.17E+08	1.17E+08	0	CD58	NM_001779	2	-	ex2	6	6
CD58_ex1	1	1.17E+08	1.17E+08	0	CD58	NM_001779	2	-	ex1	6	6
NOTCH2_ex34	1	1.2E+08	1.2E+08	0	NOTCH2	NM_024408	3	-	ex34	4	34
NOTCH2_ex27-28	1	1.2E+08	1.2E+08	0	NOTCH2	NM_024408	3	-	ex27-28	4	34
NOTCH2_ex26	1	1.2E+08	1.2E+08	0	NOTCH2	NM_024408	3	-	ex26	4	34
ITPKB_ex6	1	2.27E+08	2.27E+08	0	ITPKB	NM_002221	3	-	ex6	5	8
ITPKB_ex5	1	2.27E+08	2.27E+08	0	ITPKB	NM_002221	3	-	ex5	5	8
ITPKB_ex4	1	2.27E+08	2.27E+08	0	ITPKB	NM_002221	3	-	ex4	5	8
ITPKB_ex3	1	2.27E+08	2.27E+08	0	ITPKB	NM_002221	3	-	ex3	5	8
ITPKB_ex2	1	2.27E+08	2.27E+08	0	ITPKB	NM_002221	3	-	ex2	5	8
DNMT3A_ex18	2	25457117	25457400	0	DNMT3A	NM_022552	4	-	ex23	15	23
DNMT3A_ex17	2	25458455	25458791	0	DNMT3A	NM_022552	4	-	ex22	15	23
DNMT3A_ex16	2	25459684	25459994	0	DNMT3A	NM_022552	4	-	ex21	15	23
DNMT3A_ex15	2	25461930	25462183	0	DNMT3A	NM_022552	4	-	ex20	15	23
DNMT3A_ex13-14	2	25463061	25463710	0	DNMT3A	NM_022552	4	-	ex18-19	15	23
DNMT3A_ex12	2	25464321	25464688	0	DNMT3A	NM_022552	4	-	ex17	15	23
DNMT3A_ex9-11	2	25466655	25467631	0	DNMT3A	NM_022552	4	-	ex14-16	15	23
DNMT3A_ex8	2	25468012	25468286	0	DNMT3A	NM_022552	4	-	ex13	15	23
DNMT3A_ex6-7	2	25468778	25469289	0	DNMT3A	NM_022552	4	-	ex11-12	15	23
DNMT3A_ex5	2	25469379	25469766	0	DNMT3A	NM_022552	4	-	ex10	15	23
DNMT3A_ex4	2	25469810	25470137	0	DNMT3A	NM_022552	4	-	ex9	15	23
XPO1_ex18	2	61715613	61715954	0	XPO1	NM_003400	3	-	ex18	5	25
XPO1_ex17	2	61717667	61718021	0	XPO1	NM_003400	3	-	ex17	5	25
XPO1_ex14-16	2	61719059	61719728	0	XPO1	NM_003400	3	-	ex14-16	5	25
MAL_ex1	2	95691428	95691741	1							
MAL_ex2	2	95713593	95713982	1							
MAL_ex3	2	95715216	95715561	1							
MAL_ex4	2	95719016	95719311	1							
CXCR4_ex2	2	1.37E+08	1.37E+08	0	CXCR4	NM_001008540	2	-	ex1	1	1

CXCR4_ex1	2	1.37E+08	1.37E+08	0	CXCR4	NM_001008540	2	-	up	1	1
SF3B1_ex15-16	2	1.98E+08	1.98E+08	0	SF3B1	NM_012433	3	-	ex15-16	4	25
SF3B1_ex13-14	2	1.98E+08	1.98E+08	0	SF3B1	NM_012433	3	-	ex13-14	4	25
CD28_ex2	2	2.05E+08	2.05E+08	0	CD28	NM_006139	3	+	ex2	2	4
CD28_ex4	2	2.05E+08	2.05E+08	0	CD28	NM_006139	3	+	ex4	2	4
INPP5D_ex1	2	2.34E+08	2.34E+08	0	INPP5D	NM_001017915	2	+	ex1	17	28
INPP5D_ex2	2	2.34E+08	2.34E+08	0	INPP5D	NM_001017915	2	+	ex2	17	28
INPP5D_ex3	2	2.34E+08	2.34E+08	0	INPP5D	NM_001017915	2	+	ex3	17	28
INPP5D_ex4	2	2.34E+08	2.34E+08	0	INPP5D	NM_001017915	2	+	ex4	17	28
INPP5D_ex5	2	2.34E+08	2.34E+08	0	INPP5D	NM_001017915	2	+	ex5	17	28
INPP5D_ex6-8	2	2.34E+08	2.34E+08	0	INPP5D	NM_001017915	2	+	ex6-8	17	28
INPP5D_ex13	2	2.34E+08	2.34E+08	0	INPP5D	NM_001017915	2	+	ex13	17	28
INPP5D_ex14	2	2.34E+08	2.34E+08	0	INPP5D	NM_001017915	2	+	ex14	17	28
INPP5D_ex15	2	2.34E+08	2.34E+08	0	INPP5D	NM_001017915	2	+	ex15	17	28
INPP5D_ex16	2	2.34E+08	2.34E+08	0	INPP5D	NM_001017915	2	+	ex16	17	28
INPP5D_ex17	2	2.34E+08	2.34E+08	0	INPP5D	NM_001017915	2	+	ex17	17	28
INPP5D_ex18	2	2.34E+08	2.34E+08	0	INPP5D	NM_001017915	2	+	ex18	17	28
INPP5D_ex19	2	2.34E+08	2.34E+08	0	INPP5D	NM_001017915	2	+	ex19	17	28
INPP5D_ex20	2	2.34E+08	2.34E+08	0	INPP5D	NM_001017915	2	+	ex20	17	28
INPP5D_ex21	2	2.34E+08	2.34E+08	0	INPP5D	NM_001017915	2	+	ex21	17	28
MYD88_ex1	3	38180043	38180630	0	MYD88	NM_002468	4	+	ex1	5	5
MYD88_ex2	3	38181245	38181599	0	MYD88	NM_002468	4	+	ex2	5	5
MYD88_ex3	3	38181767	38182451	0	MYD88	NM_002468	4	+	ex3-4	5	5
MYD88_ex4	3	38182513	38182887	0	MYD88	NM_002468	4	+	ex5	5	5
SETD2_ex21	3	47058533	47058783	0	SETD2	NM_014159	6	-	ex21	21	21
SETD2_ex20	3	47059061	47059298	0	SETD2	NM_014159	6	-	ex20	21	21
SETD2_ex19	3	47061186	47061408	0	SETD2	NM_014159	6	-	ex19	21	21
SETD2_ex18	3	47079103	47079321	0	SETD2	NM_014159	6	-	ex18	21	21
SETD2_ex17	3	47084041	47084296	0	SETD2	NM_014159	6	-	ex17	21	21
SETD2_ex16	3	47087924	47088163	0	SETD2	NM_014159	6	-	ex16	21	21
SETD2_ex15	3	47098286	47099005	0	SETD2	NM_014159	6	-	ex15	21	21
SETD2_ex14	3	47103625	47103859	0	SETD2	NM_014159	6	-	ex14	21	21
SETD2_ex13	3	47108490	47108679	0	SETD2	NM_014159	6	-	ex13	21	21
SETD2_ex12	3	47125158	47125900	0	SETD2	NM_014159	6	-	ex12	21	21
SETD2_ex11	3	47127674	47127924	0	SETD2	NM_014159	6	-	ex11	21	21

SETD2_ex10	3	47129549	47129788	0	SETD2	NM_014159	6	-	ex10	21	21
SETD2_ex9	3	47139388	47139627	0	SETD2	NM_014159	6	-	ex9	21	21
SETD2_ex8	3	47142911	47143091	0	SETD2	NM_014159	6	-	ex8	21	21
SETD2_ex7	3	47144780	47144969	0	SETD2	NM_014159	6	-	ex7	21	21
SETD2_ex6	3	47147429	47147668	0	SETD2	NM_014159	6	-	ex6	21	21
SETD2_ex5	3	47155330	47155599	0	SETD2	NM_014159	6	-	ex5	21	21
SETD2_ex4	3	47158059	47158338	0	SETD2	NM_014159	6	-	ex4	21	21
SETD2_ex3	3	47161603	47166092	0	SETD2	NM_014159	6	-	ex3	21	21
SETD2_ex2	3	47168051	47168240	0	SETD2	NM_014159	6	-	ex2	21	21
SETD2_ex1	3	47205283	47205500	0	SETD2	NM_014159	6	-	ex1	21	21
RHOA_ex5	3	49397603	49397862	0	RHOA	NM_001313941	1	-	ex6	4	6
RHOA_ex4	3	49399874	49400100	0	RHOA	NM_001313941	1	-	ex5	4	6
RHOA_ex3	3	49405802	49406041	0	RHOA	NM_001313941	1	-	ex4	4	6
RHOA_ex2	3	49412831	49413074	0	RHOA	NM_001313941	1	-	ex3	4	6
TBL1XR1_ex16	3	1.77E+08	1.77E+08	0	TBL1XR1	NM_001321194	1	-	ex17	14	17
TBL1XR1_ex15	3	1.77E+08	1.77E+08	0	TBL1XR1	NM_001321194	1	-	ex16	14	17
TBL1XR1_ex14	3	1.77E+08	1.77E+08	0	TBL1XR1	NM_001321194	1	-	ex15	14	17
TBL1XR1_ex13	3	1.77E+08	1.77E+08	0	TBL1XR1	NM_001321194	1	-	ex14	14	17
TBL1XR1_ex11-12	3	1.77E+08	1.77E+08	0	TBL1XR1	NM_001321194	1	-	ex12-13	14	17
TBL1XR1_ex10	3	1.77E+08	1.77E+08	0	TBL1XR1	NM_001321194	1	-	ex11	14	17
TBL1XR1_ex8-9	3	1.77E+08	1.77E+08	0	TBL1XR1	NM_001321194	1	-	ex9-10	14	17
TBL1XR1_ex7	3	1.77E+08	1.77E+08	0	TBL1XR1	NM_001321194	1	-	ex8	14	17
TBL1XR1_ex6	3	1.77E+08	1.77E+08	0	TBL1XR1	NM_001321194	1	-	ex7	14	17
TBL1XR1_ex5	3	1.77E+08	1.77E+08	0	TBL1XR1	NM_001321194	1	-	ex6	14	17
TBL1XR1_ex4	3	1.77E+08	1.77E+08	0	TBL1XR1	NM_001321194	1	-	ex5	14	17
TBL1XR1_ex3	3	1.77E+08	1.77E+08	0	TBL1XR1	NM_001321194	1	-	ex4	14	17
BCL6_ex10	3	1.87E+08	1.87E+08	0	BCL6	NM_001706	4	-	ex10	8	10
BCL6_ex9	3	1.87E+08	1.87E+08	0	BCL6	NM_001706	4	-	ex9	8	10
BCL6_ex8	3	1.87E+08	1.87E+08	0	BCL6	NM_001706	4	-	ex8	8	10
BCL6_ex7	3	1.87E+08	1.87E+08	0	BCL6	NM_001706	4	-	ex7	8	10
BCL6_ex6	3	1.87E+08	1.87E+08	0	BCL6	NM_001706	4	-	ex6	8	10
BCL6_ex5	3	1.87E+08	1.87E+08	0	BCL6	NM_001706	4	-	ex5	8	10
BCL6_ex4	3	1.87E+08	1.87E+08	0	BCL6	NM_001706	4	-	ex4	8	10
BCL6_ex3	3	1.87E+08	1.87E+08	0	BCL6	NM_001706	4	-	ex3	8	10
TP63_ex2	3	1.9E+08	1.9E+08	0	TP63	NM_003722	4	+	ex4	11	14
TP63_ex3	3	1.9E+08	1.9E+08	0	TP63	NM_003722	4	+	ex5	11	14
TP63_ex4	3	1.9E+08	1.9E+08	0	TP63	NM_003722	4	+	ex6	11	14

TP63_ex5	3	1.9E+08	1.9E+08	0	TP63	NM_003722	4	+	ex7	11	14
TP63_ex6	3	1.9E+08	1.9E+08	0	TP63	NM_003722	4	+	ex8	11	14
TP63_ex7	3	1.9E+08	1.9E+08	0	TP63	NM_003722	4	+	ex9	11	14
TP63_ex8	3	1.9E+08	1.9E+08	0	TP63	NM_003722	4	+	ex10	11	14
TP63_ex9	3	1.9E+08	1.9E+08	0	TP63	NM_003722	4	+	ex11	11	14
TP63_ex10	3	1.9E+08	1.9E+08	0	TP63	NM_003722	4	+	ex12	11	14
TP63_ex11	3	1.9E+08	1.9E+08	0	TP63	NM_003722	4	+	ex13	11	14
TP63_ex12	3	1.9E+08	1.9E+08	0	TP63	NM_003722	4	+	ex14	11	14
TET2_ex3	4	1.06E+08	1.06E+08	0	TET2	NM_001127208	2	+	ex3	9	11
TET2_ex4	4	1.06E+08	1.06E+08	0	TET2	NM_001127208	2	+	ex4	9	11
TET2_ex5	4	1.06E+08	1.06E+08	0	TET2	NM_001127208	2	+	ex5	9	11
TET2_ex6	4	1.06E+08	1.06E+08	0	TET2	NM_001127208	2	+	ex6	9	11
TET2_ex7	4	1.06E+08	1.06E+08	0	TET2	NM_001127208	2	+	ex7	9	11
TET2_ex8	4	1.06E+08	1.06E+08	0	TET2	NM_001127208	2	+	ex8	9	11
TET2_ex9	4	1.06E+08	1.06E+08	0	TET2	NM_001127208	2	+	ex9	9	11
TET2_ex10	4	1.06E+08	1.06E+08	0	TET2	NM_001127208	2	+	ex10	9	11
TET2_ex11_1	4	1.06E+08	1.06E+08	0	TET2	NM_001127208	2	+	ex11_1	9	11
TET2_ex11_2	4	1.06E+08	1.06E+08	0	TET2	NM_001127208	2	+	ex11_2	9	11
FAT1_ex27	4	1.88E+08	1.88E+08	0	FAT1	NM_005245	3	-	ex27	26	27
FAT1_ex26	4	1.88E+08	1.88E+08	0	FAT1	NM_005245	3	-	ex26	26	27
FAT1_ex25	4	1.88E+08	1.88E+08	0	FAT1	NM_005245	3	-	ex25	26	27
FAT1_ex23-24	4	1.88E+08	1.88E+08	0	FAT1	NM_005245	3	-	ex23-24	26	27
FAT1_ex22	4	1.88E+08	1.88E+08	0	FAT1	NM_005245	3	-	ex22	26	27
FAT1_ex21	4	1.88E+08	1.88E+08	0	FAT1	NM_005245	3	-	ex21	26	27
FAT1_ex19-20	4	1.88E+08	1.88E+08	0	FAT1	NM_005245	3	-	ex19-20	26	27
FAT1_ex18	4	1.88E+08	1.88E+08	0	FAT1	NM_005245	3	-	ex18	26	27
FAT1_ex17	4	1.88E+08	1.88E+08	0	FAT1	NM_005245	3	-	ex17	26	27
FAT1_ex16	4	1.88E+08	1.88E+08	0	FAT1	NM_005245	3	-	ex16	26	27
FAT1_ex15	4	1.88E+08	1.88E+08	0	FAT1	NM_005245	3	-	ex15	26	27
FAT1_ex14	4	1.88E+08	1.88E+08	0	FAT1	NM_005245	3	-	ex14	26	27
FAT1_ex13	4	1.88E+08	1.88E+08	0	FAT1	NM_005245	3	-	ex13	26	27
FAT1_ex12	4	1.88E+08	1.88E+08	0	FAT1	NM_005245	3	-	ex12	26	27
FAT1_ex11	4	1.88E+08	1.88E+08	0	FAT1	NM_005245	3	-	ex11	26	27
FAT1_ex10	4	1.88E+08	1.88E+08	0	FAT1	NM_005245	3	-	ex10	26	27
FAT1_ex8-9	4	1.88E+08	1.88E+08	0	FAT1	NM_005245	3	-	ex8-9	26	27
FAT1_ex7	4	1.88E+08	1.88E+08	0	FAT1	NM_005245	3	-	ex7	26	27
FAT1_ex6	4	1.88E+08	1.88E+08	0	FAT1	NM_005245	3	-	ex6	26	27
FAT1_ex5	4	1.88E+08	1.88E+08	0	FAT1	NM_005245	3	-	ex5	26	27
FAT1_ex4	4	1.88E+08	1.88E+08	0	FAT1	NM_005245	3	-	ex4	26	27
FAT1_ex3	4	1.88E+08	1.88E+08	0	FAT1	NM_005245	3	-	ex3	26	27
FAT1_ex2	4	1.88E+08	1.88E+08	0	FAT1	NM_005245	3	-	ex2	26	27

IRF4_ex2	6	392983	393478	0	IRF4	NM_002460	3	+	ex2	8	9
IRF4_ex3	6	394711	395118	0	IRF4	NM_002460	3	+	ex3	8	9
IRF4_ex4	6	395737	396046	0	IRF4	NM_002460	3	+	ex4	8	9
IRF4_ex5	6	396998	397363	0	IRF4	NM_002460	3	+	ex5	8	9
IRF4_ex6	6	398718	399045	0	IRF4	NM_002460	3	+	ex6	8	9
IRF4_ex7	6	401314	401887	0	IRF4	NM_002460	3	+	ex7	8	9
IRF4_ex8	6	404908	405241	0	IRF4	NM_002460	3	+	ex8	8	9
IRF4_ex9	6	407345	407708	0	IRF4	NM_002460	3	+	ex9	8	9
HIST1H1C_ex1	6	26055967	26056698	0	HIST1H1C	NM_005319	4	-	ex1	1	1
HIST1H1B_ex1	6	27834569	27835366	0	HIST1H1B	NM_005322	3	-	ex_1	1	1
PIM1_ex1	6	37137904	37138229	0	PIM1	NM_001243186	1	+	ex1_1	6	6
PIM1_ex1-4	6	37138267	37139378	0	PIM1	NM_001243186	1	+	ex1_2-4	6	6
PIM1_ex5	6	37140661	37141059	0	PIM1	NM_001243186	1	+	ex5	6	6
PIM1_ex6	6	37141600	37141977	0	PIM1	NM_001243186	1	+	ex6	6	6
CCND3_ex5	6	41903567	41903956	0	CCND3	NM_001760	4	-	ex5	5	5
CCND3_ex4	6	41904187	41904544	0	CCND3	NM_001760	4	-	ex4	5	5
CCND3_ex3	6	41904863	41905243	0	CCND3	NM_001760	4	-	ex3	5	5
CCND3_ex2	6	41907997	41908434	0	CCND3	NM_001760	4	-	ex2	5	5
CCND3_ex1	6	41909080	41909482	0	CCND3	NM_001760	4	-	ex1	5	5
NFKBIE_ex6	6	44226846	44227220	0	NFKBIE	NM_004556	2	-	ex6	6	6
NFKBIE_ex4-5	6	44227677	44228386	0	NFKBIE	NM_004556	2	-	ex4-5	6	6
NFKBIE_ex3	6	44229258	44229695	0	NFKBIE	NM_004556	2	-	ex3	6	6
NFKBIE_ex2	6	44230187	44230510	0	NFKBIE	NM_004556	2	-	ex2	6	6
NFKBIE_ex1	6	44232609	44233631	0	NFKBIE	NM_004556	2	-	ex1	6	6
PRDM1_ex1	6	1.07E+08	1.07E+08	0	PRDM1	NM_001198	3	+	ex1	7	7
PRDM1_ex2	6	1.07E+08	1.07E+08	0	PRDM1	NM_001198	3	+	ex2	7	7
PRDM1_ex3	6	1.07E+08	1.07E+08	0	PRDM1	NM_001198	3	+	ex3	7	7
PRDM1_ex4	6	1.07E+08	1.07E+08	0	PRDM1	NM_001198	3	+	ex4	7	7
PRDM1_ex5	6	1.07E+08	1.07E+08	0	PRDM1	NM_001198	3	+	ex5	7	7
PRDM1_ex6	6	1.07E+08	1.07E+08	0	PRDM1	NM_001198	3	+	ex6	7	7
PRDM1_ex7	6	1.07E+08	1.07E+08	0	PRDM1	NM_001198	3	+	ex7	7	7
TNFAIP3_ex2	6	1.38E+08	1.38E+08	0	TNFAIP3	NM_001270508	1	+	ex2	8	9
TNFAIP3_ex3	6	1.38E+08	1.38E+08	0	TNFAIP3	NM_001270508	1	+	ex3	8	9
TNFAIP3_ex4-5	6	1.38E+08	1.38E+08	0	TNFAIP3	NM_001270508	1	+	ex4-5	8	9
TNFAIP3_ex6	6	1.38E+08	1.38E+08	0	TNFAIP3	NM_001270508	1	+	ex6	8	9
TNFAIP3_ex7	6	1.38E+08	1.38E+08	0	TNFAIP3	NM_001270508	1	+	ex7	8	9
TNFAIP3_ex8	6	1.38E+08	1.38E+08	0	TNFAIP3	NM_001270508	1	+	ex8	8	9
TNFAIP3_ex9	6	1.38E+08	1.38E+08	0	TNFAIP3	NM_001270508	1	+	ex9	8	9
CARD11_ex15	7	2963757	2964115	0	CARD11	NM_032415	5	-	ex15	8	25
CARD11_ex10	7	2973976	2974374	0	CARD11	NM_032415	5	-	ex10	8	25

CARD11_ex9	7	2976560	2976979	0	CARD11	NM_032415	5	-	ex9	8	25
CARD11_ex8	7	2977431	2977776	0	CARD11	NM_032415	5	-	ex8	8	25
CARD11_ex7	7	2978202	2978576	0	CARD11	NM_032415	5	-	ex7	8	25
CARD11_ex6	7	2979272	2979676	0	CARD11	NM_032415	5	-	ex6	8	25
CARD11_ex5	7	2983735	2984278	0	CARD11	NM_032415	5	-	ex5	8	25
CARD11_ex4	7	2985236	2985701	0	CARD11	NM_032415	5	-	ex4	8	25
BRAF_ex15	7	1.4E+08	1.4E+08	1	BRAF	NM_004333	4	-	ex15	0	18
EZH2_ex17	7	1.49E+08	1.49E+08	0	EZH2	NM_004456	4	-	ex18	3	20
EZH2_ex16	7	1.49E+08	1.49E+08	0	EZH2	NM_004456	4	-	ex17	3	20
EZH2_ex15	7	1.49E+08	1.49E+08	0	EZH2	NM_004456	4	-	ex16	3	20
MFHAS1_ex1	8	8747450	8750689	0	MFHAS 1	NM_004225	2	-	ex1	1	3
MYC_ex1	8	1.29E+08	1.29E+08	0	MYC	NM_002467	4	+	ex1	3	3
MYC_ex2	8	1.29E+08	1.29E+08	0	MYC	NM_002467	4	+	ex2	3	3
MYC_ex3	8	1.29E+08	1.29E+08	0	MYC	NM_002467	4	+	ex3	3	3
PTPRD_ex46_2	9	8314135	8315620	0	PTPRD	NM_002839	3	-	3utr	35	46
PTPRD_ex46_1	9	8315671	8318119	0	PTPRD	NM_002839	3	-	ex46	35	46
PTPRD_ex45	9	8319721	8320076	0	PTPRD	NM_002839	3	-	ex45	35	46
PTPRD_ex44	9	8331472	8331846	0	PTPRD	NM_002839	3	-	ex44	35	46
PTPRD_ex43	9	8338852	8339114	0	PTPRD	NM_002839	3	-	ex43	35	46
PTPRD_ex42	9	8340233	8340580	0	PTPRD	NM_002839	3	-	ex42	35	46
PTPRD_ex41	9	8340980	8341378	0	PTPRD	NM_002839	3	-	ex41	35	46
PTPRD_ex40	9	8341582	8342089	0	PTPRD	NM_002839	3	-	ex40	35	46
PTPRD_ex39	9	8375826	8376200	0	PTPRD	NM_002839	3	-	ex39	35	46
PTPRD_ex38	9	8376497	8376836	0	PTPRD	NM_002839	3	-	ex38	35	46
PTPRD_ex37	9	8389122	8389517	0	PTPRD	NM_002839	3	-	ex37	35	46
PTPRD_ex36	9	8404427	8404770	0	PTPRD	NM_002839	3	-	ex36	35	46
PTPRD_ex35	9	8436482	8436799	0	PTPRD	NM_002839	3	-	ex35	35	46
PTPRD_in34	9	8437141	8437278	0	PTPRD	NM_002839	3	-	in34	35	46
PTPRD_ex34	9	8449615	8449948	0	PTPRD	NM_002839	3	-	ex34	35	46
PTPRD_in33	9	8454477	8454696	0	PTPRD	NM_002839	3	-	in33	35	46
PTPRD_ex33	9	8460301	8460681	0	PTPRD	NM_002839	3	-	ex33	35	46
PTPRD_ex32	9	8465355	8465786	0	PTPRD	NM_002839	3	-	ex32	35	46
PTPRD_ex31	9	8470885	8471196	0	PTPRD	NM_002839	3	-	ex31	35	46
PTPRD_ex30	9	8484009	8484518	0	PTPRD	NM_002839	3	-	ex30	35	46
PTPRD_ex29	9	8485117	8485434	0	PTPRD	NM_002839	3	-	ex29	35	46
PTPRD_ex28	9	8485651	8486460	0	PTPRD	NM_002839	3	-	ex28	35	46
PTPRD_ex27	9	8492752	8493089	0	PTPRD	NM_002839	3	-	ex27	35	46
PTPRD_ex26	9	8497132	8497379	0	PTPRD	NM_002839	3	-	ex26	35	46
PTPRD_ex25	9	8499537	8499950	0	PTPRD	NM_002839	3	-	ex25	35	46
PTPRD_ex24	9	8500643	8501170	0	PTPRD	NM_002839	3	-	ex24	35	46
PTPRD_ex23	9	8504151	8504516	0	PTPRD	NM_002839	3	-	ex23	35	46
PTPRD_ex22	9	8507191	8507544	0	PTPRD	NM_002839	3	-	ex22	35	46
PTPRD_ex21	9	8517737	8518540	0	PTPRD	NM_002839	3	-	ex21	35	46
PTPRD_ex20	9	8521167	8521657	0	PTPRD	NM_002839	3	-	ex20	35	46
PTPRD_ex19	9	8523403	8523634	0	PTPRD	NM_002839	3	-	ex19	35	46
PTPRD_ex18	9	8524815	8525146	0	PTPRD	NM_002839	3	-	ex18	35	46
PTPRD_ex17	9	8526517	8526754	0	PTPRD	NM_002839	3	-	ex17	35	46
PTPRD_ex16	9	8527281	8527463	0	PTPRD	NM_002839	3	-	ex16	35	46
PTPRD_ex15	9	8528554	8528889	0	PTPRD	NM_002839	3	-	ex15	35	46
PTPRD_ex14	9	8633207	8633569	0	PTPRD	NM_002839	3	-	ex14	35	46
PTPRD_ex13	9	8636652	8636891	0	PTPRD	NM_002839	3	-	ex13	35	46
PTPRD_ex12	9	8733703	8733921	0	PTPRD	NM_002839	3	-	ex12	35	46

CDKN2A_ex3	9	21968118	21968351	0	CDKN2 A	NM_001195 132	1	-	3utr	4	4
CDKN2A_in2	9	21968614	21968881	0	CDKN2 A	NM_001195 132	1	-	ex3	4	4
CDKN2A_ex2	9	21970791	21971352	0	CDKN2 A	NM_001195 132	1	-	ex2	4	4
CDKN2A_in1	9	21974366	21974937	0	CDKN2 A	NM_001195 132	1	-	ex1	4	4
CDKN2A_ex1	9	21994028	21994441	0	CDKN2 A	NM_001195 132	1	-	up	4	4
CDKN2B_ex2	9	22005875	22006345	0	CDKN2 B	NM_004936	3	-	ex2	2	2
CDKN2B_ex1	9	22008686	22009063	0	CDKN2 B	NM_004936	3	-	ex1	2	2
NOTCH1_ex34	9	1.39E+08	1.39E+08	0	NOTCH 1	NM_017617	4	-	ex34	4	34
NOTCH1_ex28	9	1.39E+08	1.39E+08	0	NOTCH 1	NM_017617	4	-	ex28	4	34
NOTCH1_ex27	9	1.39E+08	1.39E+08	0	NOTCH 1	NM_017617	4	-	ex27	4	34
NOTCH1_ex26	9	1.39E+08	1.39E+08	0	NOTCH 1	NM_017617	4	-	ex26	4	34
TRAF2_ex2	9	1.4E+08	1.4E+08	0	TRAF2	NM_021138	3	+	ex2	10	11
TRAF2_ex3	9	1.4E+08	1.4E+08	0	TRAF2	NM_021138	3	+	ex3	10	11
TRAF2_ex4	9	1.4E+08	1.4E+08	0	TRAF2	NM_021138	3	+	ex4	10	11
TRAF2_ex5	9	1.4E+08	1.4E+08	0	TRAF2	NM_021138	3	+	ex5	10	11
TRAF2_ex6	9	1.4E+08	1.4E+08	0	TRAF2	NM_021138	3	+	ex6	10	11
TRAF2_ex7	9	1.4E+08	1.4E+08	0	TRAF2	NM_021138	3	+	ex7	10	11
TRAF2_ex8	9	1.4E+08	1.4E+08	0	TRAF2	NM_021138	3	+	ex8	10	11
TRAF2_ex9	9	1.4E+08	1.4E+08	0	TRAF2	NM_021138	3	+	ex9	10	11
TRAF2_ex10	9	1.4E+08	1.4E+08	0	TRAF2	NM_021138	3	+	ex10	10	11
TRAF2_ex11	9	1.4E+08	1.4E+08	0	TRAF2	NM_021138	3	+	ex11	10	11
PTEN_ex1	10	89623592	89624483	0	PTEN	NM_000314	6	+	ex1	9	9
PTEN_ex2	10	89653644	89654003	0	PTEN	NM_000314	6	+	ex2	9	9
PTEN_ex3	10	89685082	89685441	0	PTEN	NM_000314	6	+	ex3	9	9
PTEN_ex4	10	89690608	89690967	0	PTEN	NM_000314	6	+	ex4	9	9
PTEN_ex5	10	89692629	89693113	0	PTEN	NM_000314	6	+	ex5	9	9
PTEN_ex6	10	89711676	89712155	0	PTEN	NM_000314	6	+	ex6	9	9
PTEN_ex7	10	89717424	89717903	0	PTEN	NM_000314	6	+	ex7	9	9
PTEN_ex8	10	89720511	89720988	0	PTEN	NM_000314	6	+	ex8	9	9
PTEN_ex9	10	89724867	89725346	0	PTEN	NM_000314	6	+	ex9	9	9
CCND1_ex1	11	69455971	69456390	0	CCND1	NM_053056	2	+	ex1	5	5
CCND1_ex2	11	69457609	69458125	0	CCND1	NM_053056	2	+	ex2	5	5
CCND1_ex3	11	69458490	69458870	0	CCND1	NM_053056	2	+	ex3	5	5
CCND1_ex4	11	69462652	69463020	0	CCND1	NM_053056	2	+	ex4	5	5
CCND1_ex5	11	69465776	69466160	0	CCND1	NM_053056	2	+	ex5	5	5
BIRC3_ex2-3	11	1.02E+08	1.02E+08	0	BIRC3	NM_001165	4	+	ex2-3	8	9
BIRC3_ex4	11	1.02E+08	1.02E+08	0	BIRC3	NM_001165	4	+	ex4	8	9
BIRC3_ex5	11	1.02E+08	1.02E+08	0	BIRC3	NM_001165	4	+	ex5	8	9
BIRC3_ex6	11	1.02E+08	1.02E+08	0	BIRC3	NM_001165	4	+	ex6	8	9
BIRC3_ex7	11	1.02E+08	1.02E+08	0	BIRC3	NM_001165	4	+	ex7	8	9
BIRC3_ex8-9	11	1.02E+08	1.02E+08	0	BIRC3	NM_001165	4	+	ex8-9	8	9
ATM_ex57	11	1.08E+08	1.08E+08	0	ATM	NM_000051	3	+	ex57	7	63

ATM_ex58	11	1.08E+08	1.08E+08	0	ATM	NM_000051	3	+	ex58	7	63
ATM_ex59	11	1.08E+08	1.08E+08	0	ATM	NM_000051	3	+	ex59	7	63
ATM_ex60	11	1.08E+08	1.08E+08	0	ATM	NM_000051	3	+	ex60	7	63
ATM_ex61	11	1.08E+08	1.08E+08	0	ATM	NM_000051	3	+	ex61	7	63
ATM_ex62-63	11	1.08E+08	1.08E+08	0	ATM	NM_000051	3	+	ex62-63	7	63
KRAS_ex6	12	25362619	25362956	0	KRAS	NM_033360	3	-	3utr	5	6
KRAS_ex5	12	25368265	25368604	0	KRAS	NM_033360	3	-	ex5	5	6
KRAS_ex4	12	25378438	25378818	0	KRAS	NM_033360	3	-	ex4	5	6
KRAS_ex3	12	25380071	25380456	0	KRAS	NM_033360	3	-	ex3	5	6
KRAS_ex2	12	25398098	25398429	0	KRAS	NM_033360	3	-	ex2	5	6
KMT2D_ex51-54	12	49415370	49416785	0	KMT2D	NM_003482	3	-	ex51-54	54	54
KMT2D_ex49-50	12	49418245	49418844	0	KMT2D	NM_003482	3	-	ex49-50	54	54
KMT2D_ex48	12	49419806	49421245	0	KMT2D	NM_003482	3	-	ex48	54	54
KMT2D_ex46-47	12	49421507	49422100	0	KMT2D	NM_003482	3	-	ex46-47	54	54
KMT2D_ex43-45	12	49422503	49423430	0	KMT2D	NM_003482	3	-	ex43-45	54	54
KMT2D_ex35-42	12	49423894	49428875	0	KMT2D	NM_003482	3	-	ex35-42	54	54
KMT2D_ex19-34	12	49430715	49438877	0	KMT2D	NM_003482	3	-	ex19-34	54	54
KMT2D_ex15-18	12	49439671	49440656	0	KMT2D	NM_003482	3	-	ex15-18	54	54
KMT2D_ex14	12	49441620	49441979	0	KMT2D	NM_003482	3	-	ex14	54	54
KMT2D_ex12-13	12	49442280	49443124	0	KMT2D	NM_003482	3	-	ex12-13	54	54
KMT2D_ex6-11	12	49443342	49447549	0	KMT2D	NM_003482	3	-	ex6-11	54	54
KMT2D_ex1-5	12	49447648	49449232	0	KMT2D	NM_003482	3	-	ex1-5	54	54
STAT6_ex14-16	12	57492464	57493333	0	STAT6	NM_001178078	1	-	ex16-18	10	22
STAT6_ex12-13	12	57493440	57493983	0	STAT6	NM_001178078	1	-	ex14-15	10	22
STAT6_ex11	12	57495962	57496390	0	STAT6	NM_001178078	1	-	ex13	10	22
STAT6_ex10	12	57496502	57496756	0	STAT6	NM_001178078	1	-	ex12	10	22
STAT6_ex8-9	12	57498136	57498707	0	STAT6	NM_001178078	1	-	ex10-11	10	22
STAT6_in7	12	57498778	57498897	0	STAT6	NM_001178078	1	-	in9	10	22
STAT6_ex7	12	57498947	57499232	0	STAT6	NM_001178078	1	-	ex9	10	22
BTG1_ex2	12	92537806	92538333	0	BTG1	NM_001731	2	-	ex2	2	2
BTG1_ex1	12	92539054	92539331	0	BTG1	NM_001731	2	-	ex1	2	2
FOXO1_ex2	13	41133549	41135108	0	FOXO1	NM_002015	3	-	ex2	2	3
FOXO1_ex1	13	41239610	41240740	0	FOXO1	NM_002015	3	-	ex1	2	3
TRAF3_ex2	14	1.03E+08	1.03E+08	0	TRAF3	NM_145725	2	+	ex3	10	12
TRAF3_ex3	14	1.03E+08	1.03E+08	0	TRAF3	NM_145725	2	+	ex4	10	12
TRAF3_ex4	14	1.03E+08	1.03E+08	0	TRAF3	NM_145725	2	+	ex5	10	12
TRAF3_ex5	14	1.03E+08	1.03E+08	0	TRAF3	NM_145725	2	+	ex6	10	12

TRAF3_ex6	14	1.03E+08	1.03E+08	0	TRAF3	NM_145725	2	+	ex7	10	12
TRAF3_ex7	14	1.03E+08	1.03E+08	0	TRAF3	NM_145725	2	+	ex8	10	12
TRAF3_ex8	14	1.03E+08	1.03E+08	0	TRAF3	NM_145725	2	+	ex9	10	12
TRAF3_ex9	14	1.03E+08	1.03E+08	0	TRAF3	NM_145725	2	+	ex10	10	12
TRAF3_ex10	14	1.03E+08	1.03E+08	0	TRAF3	NM_145725	2	+	ex11	10	12
TRAF3_ex11	14	1.03E+08	1.03E+08	0	TRAF3	NM_145725	2	+	ex12	10	12
B2M_ex1	15	45003635	45003922	0	B2M	NM_004048	2	+	ex1	3	4
B2M_ex2	15	45007510	45007999	0	B2M	NM_004048	2	+	ex2	3	4
B2M_ex3	15	45008417	45008650	0	B2M	NM_004048	2	+	ex3	3	4
MAP2K1_ex1	15	66679454	66679971	0	MAP2K1	NM_002755	3	+	ex1	11	11
MAP2K1_ex2	15	66727255	66727686	0	MAP2K1	NM_002755	3	+	ex2	11	11
MAP2K1_ex3	15	66728963	66729341	0	MAP2K1	NM_002755	3	+	ex3	11	11
MAP2K1_ex4	15	66735516	66735805	0	MAP2K1	NM_002755	3	+	ex4	11	11
MAP2K1_ex5	15	66736884	66737243	0	MAP2K1	NM_002755	3	+	ex5	11	11
MAP2K1_ex6	15	66773983	66774328	0	MAP2K1	NM_002755	3	+	ex6	11	11
MAP2K1_ex7	15	66777218	66777640	0	MAP2K1	NM_002755	3	+	ex7	11	11
MAP2K1_ex8	15	66779456	66779724	0	MAP2K1	NM_002755	3	+	ex8	11	11
MAP2K1_ex9	15	66781443	66781724	0	MAP2K1	NM_002755	3	+	ex9	11	11
MAP2K1_ex10	15	66781946	66782211	0	MAP2K1	NM_002755	3	+	ex10	11	11
MAP2K1_ex11	15	66782730	66783063	0	MAP2K1	NM_002755	3	+	ex11	11	11
IDH2_ex2-3	15	90631709	90632089	1	IDH2	NM_002168	3	-	ex4-5	0	11
CREBBP_ex31	16	3777622	3779940	0	CREBBP	NM_004380	2	-	ex31	31	31
CREBBP_ex30	16	3781154	3781513	0	CREBBP	NM_004380	2	-	ex30	31	31
CREBBP_ex29_2	16	3781748	3781867	0	CREBBP	NM_004380	2	-	ex29_2	31	31
CREBBP_ex29_1	16	3781905	3782072	0	CREBBP	NM_004380	2	-	ex29_1	31	31
CREBBP_ex28	16	3785966	3786217	0	CREBBP	NM_004380	2	-	ex28	31	31
CREBBP_ex27	16	3786614	3786853	0	CREBBP	NM_004380	2	-	ex27	31	31
CREBBP_ex26	16	3788508	3788701	0	CREBBP	NM_004380	2	-	ex26	31	31
CREBBP_ex25	16	3789532	3789771	0	CREBBP	NM_004380	2	-	ex25	31	31
CREBBP_ex24	16	3790355	3790594	0	CREBBP	NM_004380	2	-	ex24	31	31
CREBBP_ex23	16	3794844	3795013	0	CREBBP	NM_004380	2	-	ex23	31	31
CREBBP_ex22	16	3795236	3795403	0	CREBBP	NM_004380	2	-	ex22	31	31
CREBBP_ex21	16	3799597	3799754	0	CREBBP	NM_004380	2	-	ex21	31	31
CREBBP_ex20	16	3801681	3801827	0	CREBBP	NM_004380	2	-	ex20	31	31
CREBBP_ex19	16	3807225	3807443	0	CREBBP	NM_004380	2	-	ex19	31	31
CREBBP_ex18	16	3807750	3808109	0	CREBBP	NM_004380	2	-	ex18	31	31
CREBBP_ex17	16	3808802	3809043	0	CREBBP	NM_004380	2	-	ex17	31	31
CREBBP_ex16	16	3817671	3817933	0	CREBBP	NM_004380	2	-	ex16	31	31
CREBBP_ex15	16	3819145	3819384	0	CREBBP	NM_004380	2	-	ex15	31	31
CREBBP_ex14	16	3820540	3821019	0	CREBBP	NM_004380	2	-	ex14	31	31

CREBBP_ex13	16	3823722	3823961	0	CREBBP	NM_004380	2	-	ex13	31	31
CREBBP_ex12	16	3824513	3824752	0	CREBBP	NM_004380	2	-	ex12	31	31
CREBBP_ex11	16	3827528	3827746	0	CREBBP	NM_004380	2	-	ex11	31	31
CREBBP_ex10	16	3827978	3828217	0	CREBBP	NM_004380	2	-	ex10	31	31
CREBBP_ex9	16	3828651	3828846	0	CREBBP	NM_004380	2	-	ex9	31	31
CREBBP_ex8	16	3830686	3830925	0	CREBBP	NM_004380	2	-	ex8	31	31
CREBBP_ex7	16	3831196	3831354	0	CREBBP	NM_004380	2	-	ex7	31	31
CREBBP_ex6	16	3832626	3832985	0	CREBBP	NM_004380	2	-	ex6	31	31
CREBBP_ex5	16	3841954	3842123	0	CREBBP	NM_004380	2	-	ex5	31	31
CREBBP_ex4	16	3843328	3843687	0	CREBBP	NM_004380	2	-	ex4	31	31
CREBBP_ex3	16	3860573	3860812	0	CREBBP	NM_004380	2	-	ex3	31	31
CREBBP_ex2	16	3900295	3901014	0	CREBBP	NM_004380	2	-	ex2	31	31
CREBBP_ex1	16	3929790	3929959	0	CREBBP	NM_004380	2	-	ex1	31	31
CIITA_ex1	16	10971078	10971349	0	CIITA	NM_001286402	1	+	ex1	19	20
CIITA_ex2-3	16	10989028	10989756	0	CIITA	NM_001286402	1	+	ex2-3	19	20
CIITA_ex4-5	16	10992430	10992970	0	CIITA	NM_001286402	1	+	ex4-5	19	20
CIITA_ex6	16	10995261	10995526	0	CIITA	NM_001286402	1	+	ex6	19	20
CIITA_ex7	16	10995784	10996152	0	CIITA	NM_001286402	1	+	ex7	19	20
CIITA_ex8	16	10996432	10996769	0	CIITA	NM_001286402	1	+	ex8	19	20
CIITA_ex9	16	10997478	10997862	0	CIITA	NM_001286402	1	+	ex9	19	20
CIITA_ex10	16	10998491	10998780	0	CIITA	NM_001286402	1	+	ex10	19	20
CIITA_ex11	16	11000246	11002101	0	CIITA	NM_001286402	1	+	ex11	19	20
CIITA_ex12	16	11002782	11003155	0	CIITA	NM_001286402	1	+	ex12	19	20
CIITA_ex13	16	11003935	11004226	0	CIITA	NM_001286402	1	+	ex13	19	20
CIITA_ex14	16	11009317	11009598	0	CIITA	NM_001286402	1	+	ex14	19	20
CIITA_ex15	16	11010114	11010422	0	CIITA	NM_001286402	1	+	ex15	19	20
CIITA_ex16	16	11012187	11012494	0	CIITA	NM_001286402	1	+	ex16	19	20
CIITA_ex17-18	16	11015914	11016443	0	CIITA	NM_001286402	1	+	ex17-18	19	20
CIITA_ex19	16	11016975	11017270	0	CIITA	NM_001286402	1	+	ex19	19	20
SOCS1_ex2	16	11348590	11349445	0	SOCS1	NM_003745	1	-	ex2	1	2
PLCG2_ex17	16	81941911	81942306	0	PLCG2	NM_002661	4	+	ex17	14	33
PLCG2_ex18	16	81944014	81944436	0	PLCG2	NM_002661	4	+	ex18	14	33
PLCG2_ex19	16	81946092	81946431	0	PLCG2	NM_002661	4	+	ex19	14	33
PLCG2_ex20	16	81952986	81953331	0	PLCG2	NM_002661	4	+	ex20	14	33
PLCG2_ex21	16	81954643	81954984	0	PLCG2	NM_002661	4	+	ex21	14	33
PLCG2_ex22	16	81956980	81957301	0	PLCG2	NM_002661	4	+	ex22	14	33
PLCG2_ex23	16	81960577	81960894	0	PLCG2	NM_002661	4	+	ex23	14	33

PLCG2_ex24	16	81962072	81962340	0	PLCG2	NM_002661	4	+	ex24	14	33
PLCG2_ex25	16	81964992	81965369	0	PLCG2	NM_002661	4	+	ex25	14	33
PLCG2_ex26	16	81967924	81968247	0	PLCG2	NM_002661	4	+	ex26	14	33
PLCG2_ex27	16	81969663	81970094	0	PLCG2	NM_002661	4	+	ex27	14	33
PLCG2_ex28	16	81971253	81971618	0	PLCG2	NM_002661	4	+	ex28	14	33
PLCG2_ex29	16	81972296	81972631	0	PLCG2	NM_002661	4	+	ex29	14	33
PLCG2_ex30	16	81973339	81973774	0	PLCG2	NM_002661	4	+	ex30	14	33
TP53_ex10	17	7572807	7573118	0	TP53	NM_000546	5	-	ex11	10	11
TP53_ex9	17	7573817	7574153	0	TP53	NM_000546	5	-	ex10	10	11
TP53_ex7-8	17	7576427	7577274	0	TP53	NM_000546	5	-	ex8-9	10	11
TP53_ex6	17	7577369	7577718	0	TP53	NM_000546	5	-	ex7	10	11
TP53_ex4-5	17	7578067	7578684	0	TP53	NM_000546	5	-	ex5-6	10	11
TP53_ex2-3	17	7579192	7580032	0	TP53	NM_000546	5	-	ex2-4	10	11
STAT5B_ex16	17	40359544	40359856	1	STAT5B	NM_012448	3	-	ex16	0	19
STAT3_ex21	17	40474189	40474623	0	STAT3	NM_139276	2	-	ex21	2	24
STAT3_ex20	17	40474912	40475271	0	STAT3	NM_139276	2	-	ex20	2	24
CD79B_ex4-5	17	62006476	62006946	0	CD79B	NM_001039933	2	-	ex5-6	3	6
CD79B_ex3	17	62007020	62007359	0	CD79B	NM_001039933	2	-	ex4	3	6
GNA13_ex4	17	63010263	63011060	0	GNA13	NM_006572	5	-	ex4	4	4
GNA13_ex3	17	63014261	63014532	0	GNA13	NM_006572	5	-	ex3	4	4
GNA13_ex2	17	63049534	63049956	0	GNA13	NM_006572	5	-	ex2	4	4
GNA13_in1	17	63052231	63052376	0	GNA13	NM_006572	5	-	in1	4	4
GNA13_ex1_2	17	63052410	63052734	0	GNA13	NM_006572	5	-	ex1	4	4
GNA13_ex1_1	17	63052776	63052895	0	GNA13	NM_006572	5	-	5utr	4	4
BCL2_ex3	18	60795748	60796175	0	BCL2	NM_000633	2	-	ex3	2	3
BCL2_ex2	18	60985171	60986010	0	BCL2	NM_000633	2	-	ex2	2	3
ARID3A_ex2	19	929261	929896	0	ARID3A	NM_005224	3	+	ex2	8	9
ARID3A_ex3	19	932417	932742	0	ARID3A	NM_005224	3	+	ex3	8	9
ARID3A_ex4	19	960091	960164	0	ARID3A	NM_005224	3	+	ex4	8	9
ARID3A_ex5	19	964247	964431	0	ARID3A	NM_005224	3	+	ex5	8	9
ARID3A_ex6	19	964832	965080	0	ARID3A	NM_005224	3	+	ex6	8	9
ARID3A_ex7	19	966571	966868	0	ARID3A	NM_005224	3	+	ex7	8	9
ARID3A_ex8	19	968404	968503	0	ARID3A	NM_005224	3	+	ex8	8	9
ARID3A_ex9	19	971877	972065	0	ARID3A	NM_005224	3	+	ex9	8	9
TCF3_ex19	19	1611608	1611899	0	TCF3	NM_003200	3	-	ex19	3	19
TCF3_ex18	19	1615173	1615600	0	TCF3	NM_003200	3	-	ex18	3	19
TCF3_ex17	19	1615637	1615930	0	TCF3	NM_003200	3	-	ex17	3	19
SMARCA4_ex24	19	11138459	11138626	0	SMARCA4	NM_003072	5	+	ex24	5	35
SMARCA4_ex25	19	11141405	11141569	0	SMARCA4	NM_003072	5	+	ex25	5	35
SMARCA4_ex26-28	19	11143965	11144876	0	SMARCA4	NM_003072	5	+	ex26-28	5	35
KLF2_ex1-2	19	16435625	16436955	0	KLF2	NM_016270	3	+	ex1-2	3	3
KLF2_ex3	19	16437557	16437952	0	KLF2	NM_016270	3	+	ex3	3	3
JAK3_ex24	19	17937513	17937790	0	JAK3	NM_000215	3	-	ex24	14	24
JAK3_ex23	19	17940807	17941137	0	JAK3	NM_000215	3	-	ex23	14	24
JAK3_ex22	19	17941202	17941539	0	JAK3	NM_000215	3	-	ex22	14	24
JAK3_ex21	19	17941927	17942319	0	JAK3	NM_000215	3	-	ex21	14	24
JAK3_ex20	19	17942373	17942718	0	JAK3	NM_000215	3	-	ex20	14	24

JAK3_ex18-19	19	17943218	17943848	0	JAK3	NM_000215	3	-	ex18-19	14	24
JAK3_ex15-17	19	17945268	17946136	0	JAK3	NM_000215	3	-	ex15-17	14	24
JAK3_ex14	19	17946623	17946970	0	JAK3	NM_000215	3	-	ex14	14	24
JAK3_ex13	19	17947768	17948132	0	JAK3	NM_000215	3	-	ex13	14	24
JAK3_ex11-12	19	17948657	17949269	0	JAK3	NM_000215	3	-	ex11-12	14	24
MEF2B_ex5-9	19	19256496	19258132	0	MEF2B	NM_001145785	1	-	ex5-9	8	9
MEF2B_ex4	19	19258397	19258752	0	MEF2B	NM_001145785	1	-	ex4	8	9
MEF2B_ex3	19	19259924	19260349	0	MEF2B	NM_001145785	1	-	ex3	8	9
MEF2B_ex2	19	19261367	19261654	0	MEF2B	NM_001145785	1	-	ex2	8	9
CD79A_ex4-5	19	42384627	42385157	1	CD79A	NM_001783	3	+	ex4-5	0	5
PLCG1_ex2-4	20	39788136	39788903	0	PLCG1	NM_002660	2	+	ex2-4	31	32
PLCG1_ex5-12	20	39790986	39792953	0	PLCG1	NM_002660	2	+	ex5-12	31	32
PLCG1_ex13-16	20	39793463	39794577	0	PLCG1	NM_002660	2	+	ex13-16	31	32
PLCG1_ex17-19	20	39794724	39795609	0	PLCG1	NM_002660	2	+	ex17-19	31	32
PLCG1_ex20	20	39796382	39796679	0	PLCG1	NM_002660	2	+	ex20	31	32
PLCG1_ex21-22	20	39797297	39797926	0	PLCG1	NM_002660	2	+	ex21-22	31	32
PLCG1_ex23	20	39797987	39798276	0	PLCG1	NM_002660	2	+	ex23	31	32
PLCG1_ex24	20	39798655	39799020	0	PLCG1	NM_002660	2	+	ex24	31	32
PLCG1_ex25-27	20	39800720	39801630	0	PLCG1	NM_002660	2	+	ex25-27	31	32
PLCG1_ex28-32	20	39801950	39803259	0	PLCG1	NM_002660	2	+	ex28-32	31	32
EP300_ex1	22	41488899	41489212	0	EP300	NM_001429	3	+	ex1	31	31
EP300_ex2	22	41513037	41513938	0	EP300	NM_001429	3	+	ex2	31	31
EP300_ex3	22	41521731	41522155	0	EP300	NM_001429	3	+	ex3	31	31
EP300_ex4	22	41523380	41523858	0	EP300	NM_001429	3	+	ex4	31	31
EP300_ex5	22	41525803	41526118	0	EP300	NM_001429	3	+	ex5	31	31
EP300_ex6	22	41527281	41527748	0	EP300	NM_001429	3	+	ex6	31	31
EP300_ex7	22	41531707	41532020	0	EP300	NM_001429	3	+	ex7	31	31
EP300_ex8	22	41533547	41533904	0	EP300	NM_001429	3	+	ex8	31	31
EP300_ex9	22	41536034	41536371	0	EP300	NM_001429	3	+	ex9	31	31
EP300_ex10	22	41536942	41537337	0	EP300	NM_001429	3	+	ex10	31	31
EP300_ex11	22	41542633	41542930	0	EP300	NM_001429	3	+	ex11	31	31
EP300_ex12	22	41543731	41544060	0	EP300	NM_001429	3	+	ex12	31	31
EP300_ex13	22	41544932	41545289	0	EP300	NM_001429	3	+	ex13	31	31
EP300_ex14	22	41545654	41546313	0	EP300	NM_001429	3	+	ex14	31	31
EP300_ex15-16	22	41547727	41548464	0	EP300	NM_001429	3	+	ex15-16	31	31
EP300_ex17	22	41550889	41551199	0	EP300	NM_001429	3	+	ex17	31	31
EP300_ex18	22	41553062	41553523	0	EP300	NM_001429	3	+	ex18	31	31
EP300_ex19	22	41554306	41554615	0	EP300	NM_001429	3	+	ex19	31	31
EP300_ex20	22	41556526	41556836	0	EP300	NM_001429	3	+	ex20	31	31
EP300_ex21	22	41558617	41558902	0	EP300	NM_001429	3	+	ex21	31	31

EP300_ex22	22	41559947	41560244	0	EP300	NM_001429	3	+	ex22	31	31
EP300_ex23	22	41562493	41562780	0	EP300	NM_001429	3	+	ex23	31	31
EP300_ex24-25	22	41564343	41564982	0	EP300	NM_001429	3	+	ex24-25	31	31
EP300_ex26	22	41565397	41565730	0	EP300	NM_001429	3	+	ex26	31	31
EP300_ex27	22	41566300	41566685	0	EP300	NM_001429	3	+	ex27	31	31
EP300_ex28	22	41568392	41568778	0	EP300	NM_001429	3	+	ex28	31	31
EP300_ex29	22	41569517	41569863	0	EP300	NM_001429	3	+	ex29	31	31
EP300_ex30-31	22	41572202	41575072	0	EP300	NM_001429	3	+	ex30-31	31	31
NLGN4X_ex6	X	5810803	5811762	1							
NLGN4X_ex5	X	5821093	5821932	1							
NLGN4X_ex4	X	5827068	5827307	1							
NLGN4X_ex3	X	5947278	5947517	1							
NLGN4X_ex2	X	6069035	6069514	1							
BCOR_ex15	X	39911252	39911763	0	BCOR	NM_001123385	1	-	ex15	14	15
BCOR_ex13-14	X	39913028	39913697	0	BCOR	NM_001123385	1	-	ex13-14	14	15
BCOR_ex12	X	39914511	39914876	0	BCOR	NM_001123385	1	-	ex12	14	15
BCOR_ex11	X	39916298	39916684	0	BCOR	NM_001123385	1	-	ex11	14	15
BCOR_ex10	X	39921204	39921756	0	BCOR	NM_001123385	1	-	ex10	14	15
BCOR_ex9	X	39921888	39922435	0	BCOR	NM_001123385	1	-	ex9	14	15
BCOR_ex8	X	39922750	39923214	0	BCOR	NM_001123385	1	-	ex8	14	15
BCOR_ex7	X	39923479	39923962	0	BCOR	NM_001123385	1	-	ex7	14	15
BCOR_ex6	X	39930116	39930523	0	BCOR	NM_001123385	1	-	ex6	14	15
BCOR_ex5	X	39930780	39931053	0	BCOR	NM_001123385	1	-	ex5	14	15
BCOR_ex4	X	39931492	39934547	0	BCOR	NM_001123385	1	-	ex4	14	15
BCOR_ex3	X	39935597	39935896	0	BCOR	NM_001123385	1	-	ex3	14	15
BCOR_ex2	X	39936987	39937292	0	BCOR	NM_001123385	1	-	ex2	14	15
DDX3X_ex1	X	41193396	41193635	0	DDX3X	NM_001356	4	+	ex1	17	17
DDX3X_ex2	X	41196595	41196784	0	DDX3X	NM_001356	4	+	ex2	17	17
DDX3X_ex3	X	41198218	41198407	0	DDX3X	NM_001356	4	+	ex3	17	17
DDX3X_ex4	X	41200683	41200922	0	DDX3X	NM_001356	4	+	ex4	17	17
DDX3X_ex5-6	X	41201708	41202099	0	DDX3X	NM_001356	4	+	ex5-6	17	17
DDX3X_ex7	X	41202416	41202655	0	DDX3X	NM_001356	4	+	ex7	17	17
DDX3X_ex8	X	41202924	41203127	0	DDX3X	NM_001356	4	+	ex8	17	17
DDX3X_ex9-10	X	41203273	41203691	0	DDX3X	NM_001356	4	+	ex9-10	17	17
DDX3X_ex11-12	X	41204385	41204837	0	DDX3X	NM_001356	4	+	ex11-12	17	17
DDX3X_ex13-14	X	41205453	41205876	0	DDX3X	NM_001356	4	+	ex13-14	17	17

DDX3X_ex15	X	41206069	41206308	0	DDX3X	NM_001356	4	+	ex15	17	17
DDX3X_ex16	X	41206481	41206763	0	DDX3X	NM_001356	4	+	ex16	17	17
DDX3X_ex17	X	41206838	41207027	0	DDX3X	NM_001356	4	+	ex17	17	17
BTK_ex15	X	1.01E+08	1.01E+08	0	BTK	NM_001287 344	1	-	ex15	2	19
BTK_ex2	X	1.01E+08	1.01E+08	0	BTK	NM_001287 344	1	-	ex2	2	19
SRY_ex1	Y	2654891	2655790	1							
